# Four new species of the whalefish genus *Gyrinomimus* Parr, 1934, with the establishment of three new species groups (Beryciformes, Cetomimidae)

**DOI:** 10.3897/zookeys.1279.171946

**Published:** 2026-05-05

**Authors:** Yo Su, John R. Paxton, Hsuan-Ching Ho

**Affiliations:** 1 International Doctoral Program of Marine Science and Technology, National Sun Yat-sen University, Kaohsiung, Taiwan National Kaohsiung University of Science and Technology Kaohsiung Taiwan; 2 Department and Graduate Institution of Aquaculture, National Kaohsiung University of Science and Technology, Kaohsiung, Taiwan National Sun Yat-sen University Kaohsiung Taiwan; 3 Australian Museum, Sydney, New South Wales, Australia National Museum of Marine Biology & Aquarium Checheng Taiwan; 4 Taiwan Ocean Research Institute, National Institutes of Applied Research, Kaohsiung, Taiwan Australian Museum Sydney Australia; 5 National Museum of Marine Biology & Aquarium, Checheng, Pingtung, Taiwan Taiwan Ocean Research Institute, National Institutes of Applied Research Kaohsiung Taiwan

**Keywords:** Biodiversity, deep sea, ichthyology, morphology, taxonomy

## Abstract

Four new species of the flabby whalefish genus *Gyrinomimus* Parr, 1934 are described based on female specimens: *G. alepis***sp. nov**. from the southern Atlantic, *G. amaokai***sp. nov**. from the Northern Hemisphere, *G. johnpaxtoni* Su & Ho, **sp. nov**. from the Pacific and Atlantic, and *G. johnsoni***sp. nov**. from the northern Pacific. Each of the new species differs from congeners in the length of gill filaments on fourth gill arch, numbers of dorsal- and anal-fin rays, gill rakers, head and lateral-line pores, anal lappets and vertebrae, distribution of cavernous tissue, and the shape and length/width ratio of copular tooth plate. Moreover, three species groups are defined: *G. bruuni* species group, *G. grahami* species group, and *G. myersi* species group. Each group differs in the length of gill filaments, numbers of anal lappet and gill rakers, and distribution of cavernous tissue. Diagnostic characters and detailed descriptions of the new species are provided and compared to congeners, with variations discussed. Finally, an identification key to species groups and all known species is provided.

## Introduction

Whalefishes of the family Cetomimidae live primarily in the bathypelagic waters of the world’s oceans, with adults generally living below 1500 meters. With tiny eyes and an extensive sensory lateral-line system on the head and body, whalefishes show striking adaptations to pelagic life in the deepest waters, including a huge mouth and dark coloration. Members of the family also exhibit an extraordinary metamorphosis and sexual dimorphism, with two previous families, Mirapinnidae and Megalomycteridae, actually representing the larval stages and males of Cetomimidae, respectively (Johnson et al. 2009). A total of 27 species in 14 genera are currently recognized as valid (Johnson et al. 2009; Paxton et al. 2016; Kobyliansky et al. 2023).

The genus *Gyrinomimus* Parr, 1934, second largest in the family after *Cetomimus* Goode & Bean, 1895, includes seven nominal species, namely, *G. andriashevi* Fedorov, Balushkin & Trunov, 1987, *G. bruuni* Rofen, 1959, *G. grahami* Richardson & Garrick, 1964, *G. myersi* Parr, 1934, *G. notius* Fedorov & Balushkin, 1983, *G. parri* Bigelow, 1961, and *G. simplex* Parr, 1946 (Paxton 1989, 2002, 2015; Paxton and Gon 1990). Of these species, *G. notius* and *G. simplex* are now regarded as junior synonyms of *G. grahami* and *G. myersi*, respectively, because teeth rows on jaws, vomer, palatine, and ectopterygoid are added with growth (Paxton 1989, 2002). The genus is characterized by having 14–21 dorsal-fin rays; 47–59 total vertebrae; 3 free gill arches, no slit behind ventral arm of fourth arch; gill rakers forming plates; jaw teeth long, their length 3 times their basal width and arranged in indistinct longitudinal rows; and vomerine tooth plate flat, rectangular, or oval (Paxton 1989).

As stated in Paxton (2002), the taxonomy of *Gyrinomimus* has been studied by himself (JP) and was nearly completed. However, the results had not been published prior to his death in 2023. With the help of JP’s family and colleagues of the Australian Museum (AMS), the first author undertook his unpublished data and continued his work. In this study, we describe four new species of *Gyrinomimus* and define three new species groups based on morphological characteristics. An identification key to species of *Gyrinomimus* is provided. Lastly, morphological characteristics and distribution pattern of *Gyrinomimus* are also discussed.

## Materials and methods

### Morphological data

Methodology and terminology follow Paxton (1989), with the following additions: the raker at angle was not included in the lower-raker count and the number of gill rakers is expressed as rakers on upper arm + raker at angle + rakers at lower arm; gill filaments were measured at the longest filament on respective gill arches. Medial rakers refer to those on the inner faces of gill arches. All measurements were made using calipers and rounded to the nearest 0.1 mm, except for lengths larger than 300 mm, which were measured using a regular ruler and rounded to the nearest 1 mm. Morphometric data are expressed as % or ratios of standard length (**SL**) and/or head length (**HL**) whenever available. Morphological data were generally taken from the left side of the specimen, unless the character is damaged and the right side was adopted and noted, except for counts of gill rakers and measurements of gill filaments, which were taken from right side. Vertebral formulae were determined using X-radiographs. Counts of pectoral-fin rays, anal lappets and both lateral-line pores and scales were taken from both sides. Counts of head pores include all openings and are abbreviated as follows: mandibular pores (**MD**); preopercular pores (**POP**); infraorbital pores (**IO**); supraorbital pores (**SO**); main-canal pores (**M**); supratemporal pores (**ST**). Other abbreviations: **GRI–III**, gill rakers (forming plates) on first to third arches; **N**, nostrils.

### Specimen depository

Specimens examined in this study are deposited at Australian Museum, Sydney, New South Wales, Australia (**AMS**); Biodiversity Research Center, Academia Sinica, Taipei, Taiwan (**ASIZP**); Royal British Columbia Museum, Victoria, Canada (**BCPM**); Marine Research Institute of Iceland, Reykjavik, Iceland (**HAF**); The Hokkaido University Museum, Hakodate, Hokkaido, Japan (**HUMZ**); Natural History Museum of Los Angeles County, Los Angeles, California, U.S.A (**LACM**); Ichthyology Department, Museum of Comparative Zoology, Harvard University, Cambridge, Massachusetts, U.S.A (**MCZ**); Muséum National d’Histoire naturelle, Paris, France (**MNHN**); Museum of New Zealand Te Papa Tongarewa, Wellington, New Zealand (**NMNZ P**); National Museum of Nature and Science, Zoology Department, Division of Fishes, Tsukuba, Japan (**NSMT-P**); Tokai Fisheries collection (**TH**), now in NSMT; Faculty of Science, Museum of Natural History, Oregon State University, Corvallis, Oregon, U.S.A. (**OS**); Marine Vertebrate Collection, Scripps Institution of Oceanography, La Jolla, California, U.S.A. (**SIO**); National Museum of Natural History, Smithsonian Institution, Washington D.C., U.S.A (**USNM**); Peabody Museum of Natural History, Division of Vertebrate Zoology, Ichthyology, Yale University, New Haven, Connecticut, U.S.A. (**YPM**); Laboratory of Ichthyology, Zoological Institute, Russian Academy of Sciences, St. Petersburg, Russia (**ZIN**); Johann Heinrich von Thünen-Institut, Bundesforschungsinstitut für Ländliche Räume, Wald und Fischerei, Institut für Seefischerei, Hamburg, Germany (**ISH**), now in Zoologisches Institut und Zoologisches Museum der Universität Hamburg, Germany (**ZMH**); and Vertebrater, Fiskesamlingen, Københavns Universitet, Zoologisk Museum, Copenhagen, Denmark. (**ZMUC P**.). All type specimens of nominal species were examined by JP, with additional specimens examined by YS and/or HCH.

### Molecular analysis

The Cytochrome c oxidase subunit I (COI) sequence of paratype of *G. johnpaxtoni* sp. nov. (LC799418; voucher: HUMZ 234625) was downloaded from GenBank (Benson et al. 2012), and the sequence of *G. bruuni* (FTWS392-09; voucher: ASIZP 69735) was downloaded from the Barcode of Life Data System (BOLD) (Ratnasingham and Hebert 2007). Genetic distance was calculated by Kimura-2-Parameter (K2P) substitution model (Kimura 1980) using the software MEGA11 (Tamura et al. 2021). Due to the paucity of genetic materials and the uncertainty on the identification of sequences on GenBank and BOLD System, we do not include all of the sequences of *Gyrinomimus* and both male and larval specimens of cetomimids for analyses.

## Results

### Family Cetomimidae

#### Gyrinomimus

Taxon classification

Animalia

CetomimiformesCetomimidae

Parr, 1934

18FE8D4F-1B30-56AB-BB5C-DD58E73AEBF2

Gyrinomimus Parr, 1934:29. Type species: Gyrinomimus myersi Parr, 1934.

##### Diagnosis.

Elongated, closely-set teeth in distinct longitudinal rows on the jaws, palatine, and ectopterygoid; free gill arches 3; tooth plates of gill arches solid, flat, and covering most of the lateral face of the arch; ventral pharyngeal tooth plates absent; total vertebrae 47–59; lateral-line scales rectangular or strap-like and curved; vomerine tooth patch rectangular or laterally elongate and flat.

##### Remarks.

Paxton (1989) stated that gill rakers are present on all four gill arches in the genus *Gyrinomimus*. However, based on our observation, most specimens lack gill rakers on fourth gill arch, with only three specimens of *G. johnsoni* sp. nov. show this character. Therefore, the statement of rakers on all four gill arches for all *Gyrinomimus* in Paxton (1989) should be revised accordingly. Based on the morphological characters, species of *Gyrinomimus* can be separated into three species groups described herein:

##### *Gyrinomimus myersi* species group.

This species group differs from the other two groups in having medial tooth plates on the first gill arch absent, anal lappets absent, cavernous tissue present at least one other area besides the anus (in 3 of the 4 species behind the pectoral girdle under the pectoral fin), and tiny to small filaments on the fourth gill arch. The group includes *G. myersi*, *G. parri*, and two potentially undescribed species.

##### *Gyrinomimus grahami* species group.

This species group differs from the other two groups in having medial tooth plates on the first gill arch present, anal lappets absent, gill filaments on the fourth gill arch tiny to small, and cavernous tissue, limited to around the anus or at most as a separate patch posterodorsal to the anus. The group includes *G. grahami*, *G. johnsoni* sp. nov., and one potentially undescribed species.

##### *Gyrinomimus bruuni* species group.

This species group differs from the other two groups in having filaments on the fourth gill arch moderate to long and having anal lappets and supported by scales or not (scales absent in *G. alepis* sp. nov.). The group includes *G. bruuni*, *G. alepis*, *G. amaokai* sp. nov., *G. johnpaxtoni* sp. nov., and one potentially undescribed species. In addition, the anal-fin base of the holotype of *G. andriashevi* is damaged, rendering the condition of the anal lappets indeterminable (Paxton and Gon 1990). Nevertheless, *G. andriashevi* is tentatively assigned to this group based on the presence of well-developed filaments on the fourth gill arch and pending further study.

### Taxonomic account

#### Gyrinomimus alepis sp. nov.

Taxon classification

Animalia

CetomimiformesCetomimidae

3F389398-4508-50FC-9F30-DDCCD13C9F0A

https://zoobank.org/CB8074AE-8318-4CC8-8D2E-F481ACFC8656

**Figure 1.**
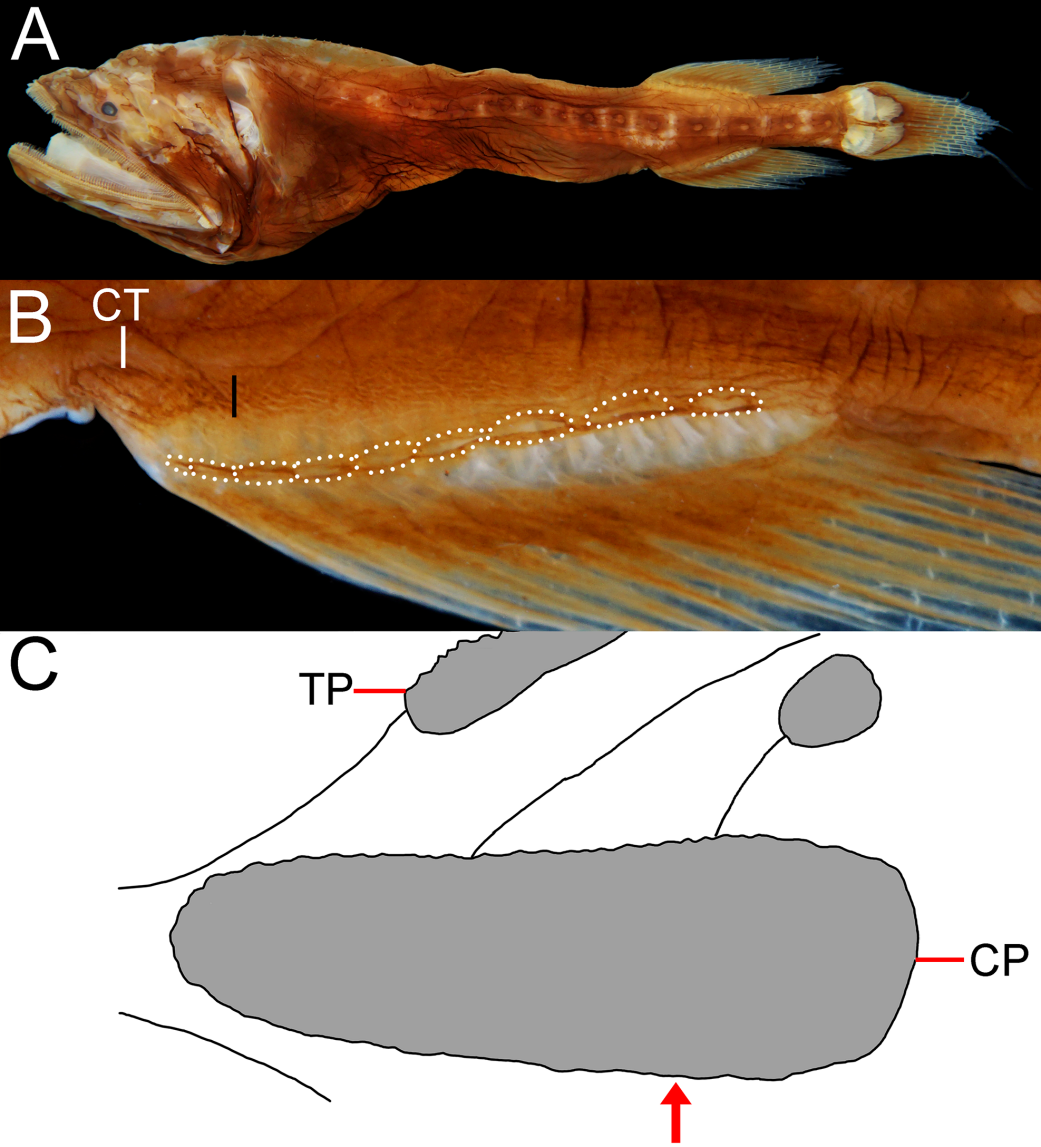
Preserved specimen of *Gyrinomimus alepis* sp. nov., holotype, ZMH-ICH-108930, 62.4 mm SL. **A**. Lateral view; **B**. Close-up image of anal-fin base, featuring cavernous tissue, with its posterior extension indicated by black bar, and anal lappets (circled); **C**. Illustration of copular tooth plates (CP) and anteriormost tooth plate (TP) on first gill arch. Anterior to left. Figures not to scale. Photo by Y.-C. Fan (**B**).

**Figure 2.**
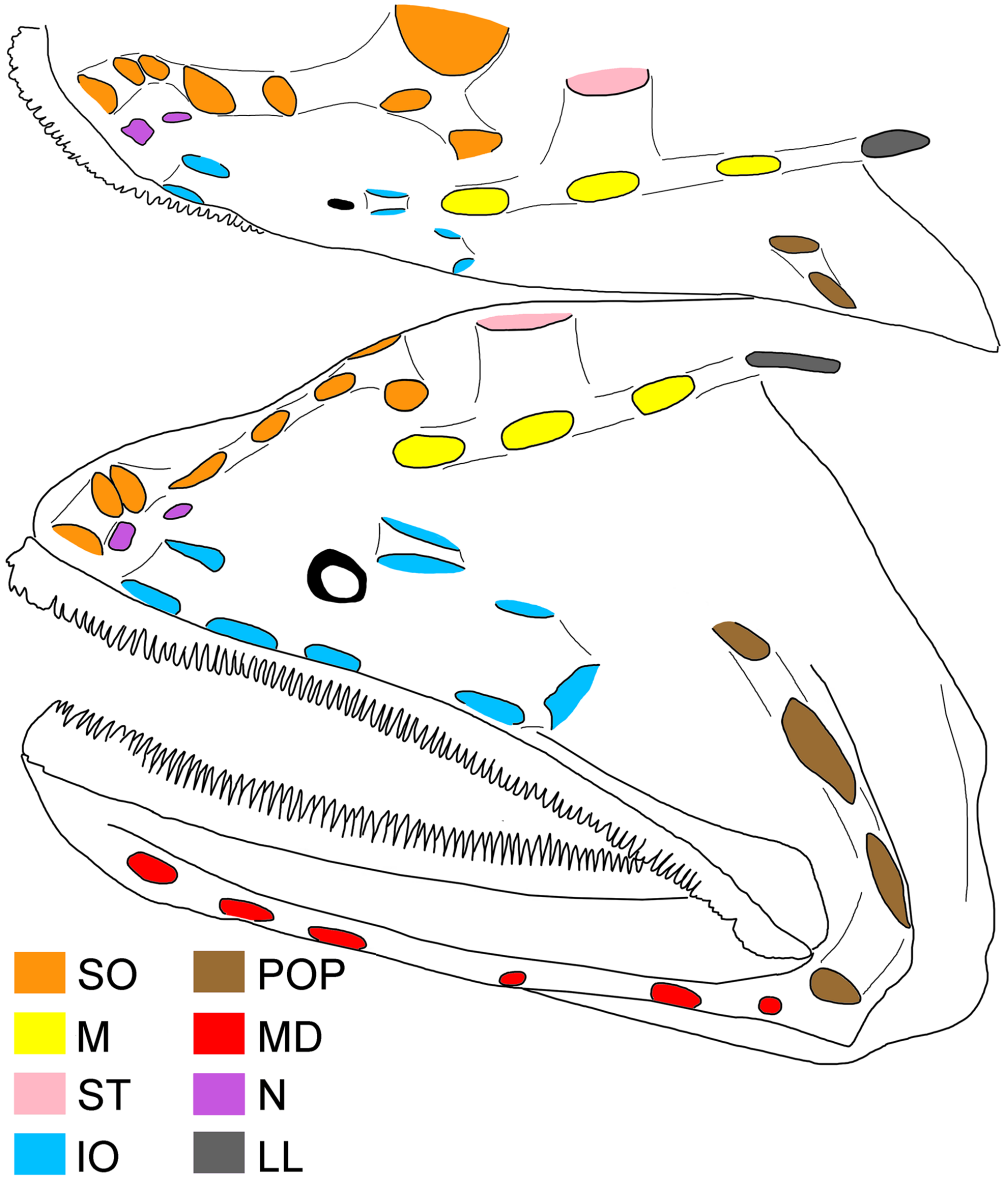
Illustration of head pores of *Gyrinomimus alepis* sp. nov., holotype, ZMH-ICH-108930, 62.4 mm SL. Upper: dorsal view; lower: lateral view. Anterior to left. Figures not to scale.

Figs 1, 2, Tables 1, 2

##### New English name.

Scaleless flabby whalefish.

**Table 1.** Meristic characters of *Gyrinomimus bruuni* species group. Paired characters are presented as left/right whenever available. Abbreviations: HT, holotype; NT, non-types; PT, paratypes.

	*G. alepis* sp. nov.	*G. andriashevi*	*G. amaokai* sp. nov.		*G. johnpaxtoni* sp. nov.		*G. bruuni*	
	This study	Paxton and Gon 1990	This study		This study		This study	
	HT	HT	HT	PT (*n* = 6)	HT	PT (*n* = 15)	HT	NT (*n* = 6)
Dorsal-fin rays	15	14	19	17–21	19	19–21	20	17–20
Pectoral-fin rays	17/17	ca 19	16/16	17–22/17–21	16/16	16–21/15–19	18/18	18–21/19–20
Anal-fin rays	15	14	19	17–20	19	18–21	20	17–20
Principal caudal-fin rays	8 + 8	–	8 + 8	7–8 + 7–8	8 + 8	7–8 + 7–8	8 + 8	7–8 + 7–8
Vertebrae	36 + 18 = 54	58 (total)	34 + 19 = 53	32–34 + 17–20 = 51–53	38 + 18 = 56	35–38 + 18–20 = 54–58 (*n* = 14)	35 + 19 = 54	35–38 + 18–20 = 54–58
Lateral-line scales	16/16	ca 16	20/19	18–22/17–19	23/22	18–24/18–21	19/ca 19	18–21/17–19
Lateral-line pores	17/17	ca 17	21/20	19–22/18–20	24/23	19–25/19–22	20/ca 20	19–22/18–20
GRI	2 + 1 + 8	1 + 1 + 2–3	1 + 1 + 2	1–3 + 1 + 2–4	1 + 1 + 2	1 + 1 + 2–3	1 + 1 + 4	1–2 + 1 + 2–4
GRII	1 + 1 + 3	–	1 + 1 + 2	1 + 1 + 2–4	1 + 1 + 2	1 + 1 + 2–4	1 + 1 + 4	0–1 + 1 + 2–4
GRIII	0 + 1 + 1	–	1 + 1 + 1	1 + 1 + 1	0 + 1 + 1	0 + 1 + 1	0 + 1 + 2	0–1 + 1 + 1–2
Medial GRI	4	0	0	0	0	0	0	0
Anal lappet	9/10	N/A	8/8	8–14/11–14	7/7	4–9/4–8	6/6	2–3/3–4
Head pores								
Mandibular	6	ca 7	9	8–9	9	8–11	9	8–9
Preopercle	4	ca 3	5	5	5	3–5	ca 4	3–5
Infraorbital	7 + 2	7 + 2	7 + 2	7 + 2 (rarely 8 + 2)	8 + 2	8 + 2	8 + 2	8 + 2 (rarely 7 + 2)
Supraorbital	8	6	7	7–8	8	7–8	8	7–8
Main canal	3	3–4	3	3–4	4	3–4	4	2–4
Supratemporal	1	1	1	1	1	1	1	1

**Table 2.** Morphometric characters of *Gyrinomimus alepis* sp. nov. and *G. andriashevi* Fedorov, Balushkin & Trunov, 1987. Abbreviations: A, anal-fin; D, dorsal-fin; HT, holotype; P, pectoral-fin.

	*G. alepis* sp. nov.	*G. andriashevi*	
	This study	Fedorov et al. 1987	Paxton and Gon 1990
	HT	HT	
SL (mm)	62.4	245	232
% SL			
HL	32.7	–	27.6
Head width	10.6	–	–
Eye diameter	1.8	1.0	–
Snout length	9.5	10.2	8.8
Upper-jaw length	28.0	23.7	26.1
Lower-jaw length	27.9	–	–
Posterior premaxilla to opercular margin	4.3	–	5.1
Body depth	23.1	20.8	–
Anus to A origin	2.1	–	–
D–A	11.4	–	12.7
Predorsal length	73.4	75.5	–
Prepectoral length	30.1	31.8	–
Preanal length	75.0	76.8	78.8
D base	13.9	13.1	–
A base	13.9	12.7	12.4
P length	7.2	2.9	–
Caudal-peduncle depth	5.8	11.4	–
Caudal-peduncle length	13.3	6.1	10.6
Copular tooth plate length	6.3	–	8.5
Copular tooth plate width	3.0	–	–
Gill filaments on first arch	1.4	–	–
Gill filaments on fourth arch	1.1	–	–
% Gill filaments on first arch			
Gill filaments on fourth arch	82.4	–	–
Length/width ratio of copular tooth plate	2.1	–	3.4

##### Holotype.

• ZMH-ICH-0108930 (formerly ISH 1190-1971), 62.4 mm SL, south Atlantic, 37°8'S, 05°23'E, 0–1900 m, FRV *Walther Herwig*, station 412-II/71, 21 Mar. 1971.

##### Diagnosis.

A species belonging to *G. bruuni* species group and differs from all congeners in having: single ridge of skin over the anal fin with incipient anal lappets without scales; filaments on fourth gill arch well developed, its length 82.4% filament on first arch; cavernous tissue present around anus and extending to base of third anal-fin ray; vertebrae 36 + 18 = 54; GRI 2 + 1 + 8; medial GRI 4; medial GRII 1; dorsal-fin rays 15; anal-fin rays 15; lateral-line pores 17; lateral-line scales 16; and copular tooth plate rather broad, greatest width 2.1 in length.

##### Description.

Meristic and morphometric characters are provided in Tables 1, 2. Dorsal-fin rays 15; pectoral-fin rays 17/17; anal-fin rays 15; principal caudal-fin rays 8 + 8. GRI 2 + 1 + 8; GRII 1 + 1 + 3; GRIII 0 + 1 + 1; medial GRI 4; medial GRII 1. Lateral-line pores 17; lateral-line scales 16. Anal lappets 9/10, without scales. Head pores (Fig. 2): MD 6; POP 4; IO 7 + 2 = 9; SO 8; M 3; ST 1. Vertebrae 36 + 18 = 54.

Body rather slender, greatest depth at pectoral-fin base over first lateral-line pore, depth 4.3 in SL, compressed. Head moderate, length 3.1 in SL, slightly depressed anteriorly, becoming broader posteriorly. Nostrils similar to head lateral-line pores, closer to tip of snout than eye; anterior nostril ~ 1/2 of posterior one; anterior nostril rounded, and posterior one horizontally oval; nasal organ mostly in anterior nostril. Eye tiny, diameter 18.4 in HL. Interorbital broadly convex, without distinct pits; anterolateral projection of frontal slightly posterior to eye. Caudal peduncle short and deep, length and depth 2.5 and 5.6 in HL, respectively. Cavernous tissue surrounding anus and extending to base of third anal-fin ray (Fig. 1B). Anal lappets rudimentary, forming very small bumps along anal-fin base; no trace of scales on anal lappets (Fig. 1B).

Mouth enormous and oblique; upper-jaw length 1.2 in HL; lower jaw with two posterior spines, with ventral spine blunt and lateral spine short, blunt, and covered under skin. Jaw teeth elongated, in distinct longitudinal rows; inner row of teeth largest; premaxillary and dentary symphyses edentate. Upper jaw with three rows of teeth anteriorly, its number slightly decreases posteriorly, with two rows posteriorly. Lower-jaw teeth similar to those of upper jaw, with four rows anteriorly and two rows posteriorly. Vomerine, palatine, and ectopterygoid teeth similar in shape to jaw teeth. Vomerine tooth patch dome shaped, with two rows of teeth. Palatine tooth patch begins at level of middle of vomer, with two rows of teeth; its end anterior to level of eye. Ectopterygoid tooth patch narrow, with about two teeth rows separated by small gap from palatine tooth patch; its origin posterior to palatine, extending posteriorly beyond rictus almost to level of end of premaxilla. Copular tooth patch stout, its anterior margin blunt, with nearly straight lateral margins, and very slightly widens posteriorly; its length and width 5.2 and 10.9 in HL, respectively; length/width ratio 2.1 (Fig. 2C).

Three free gill arches with small slit behind ventral arm of third arch near angle, extending ~1/2 length of filaments-bearing part of ventral arm; dorsal arm of arch I 1/2 free; dorsal arm of arch II 1/3 free. Gill filaments of all arches long and well developed; length of filaments on arch IV 82.4% length of filaments on arch I. Gill rakers forming plates on lateral face of outer three arches and medial face of outer two arches. Anterior margin of anteriormost hypobranchial tooth plate of first arch slightly posterior to anterior margin of copular tooth plate. Two dorsal pharyngeal tooth plates, both domed and rounded; no ventral pharyngeal tooth plate.

Lateral-line pores large, anterior pores almost as wide as canal, becoming smaller posteriorly, to ~ 1/3 of canal length. Lateral line without any flaps or keels. One to two papillae on both anterior and posterior margins of pores anterior to anal fin. Vertical row of papillae on base of caudal fin; numbers at least 16 on right side due to partial damage. Dorsal accessory line of neuromasts beginning slightly before level of upper limit of gill opening, as two parallel rows of 18 papillae, extending before mid-dorsal keel.

Lateral-line system of head well developed. Head with numerous scattered papillate superficial neuromasts, ~ 9 (partially damaged) on tip of snout between midline and first supraorbital pore; no papillae above anterior nostril; two papillae dorsal to first infraorbital pore; no papillae dorsal to second and third infraorbital pores; two papillae dorsal to second main pore; no papillae medial to supratemporal pore; 12 papillae anterior to preopercular pores; seven papillae scattered above mandibular pores.

Dorsal and anal fins far back on body, with dorsal-fin origin slightly anterior to that of anal fin; their tips pointed posteriorly. Dorsal fin with 8^th^–13^th^ rays branched, others unbranched. Anal fin with 6^th^–11^th^ rays branched, others unbranched. Pectoral fin low, broad-based, directed posterodorsally, and small, length 4.5 in HL; middle rays longest; all rays simple. No trace of subpectoral organ (sensu Paxton 1989).

##### Coloration.

When preserved (Fig. 1A), body brown with dorsal and anal fins slightly paler. Gill arches, rakers and filaments, inner face of operculum pale. Teeth transparent. Peritoneum black. Esophagus and mouth roof with dark marbled patterns.

##### Distribution.

Currently only known from the holotype collected at southern Atlantic Ocean.

##### Etymology.

The specific name is a combination of *a*, without, and *lepis*, scales. In reference to the absence of scales on the anal lappets. A noun in apposition.

#### Gyrinomimus amaokai sp. nov.

Taxon classification

Animalia

CetomimiformesCetomimidae

40189A5F-977E-5C8E-8DAA-FBDCEB6A4FC6

https://zoobank.org/7C711DD4-090E-45ED-BB08-F8D0E95A914D

**Figure 3.**
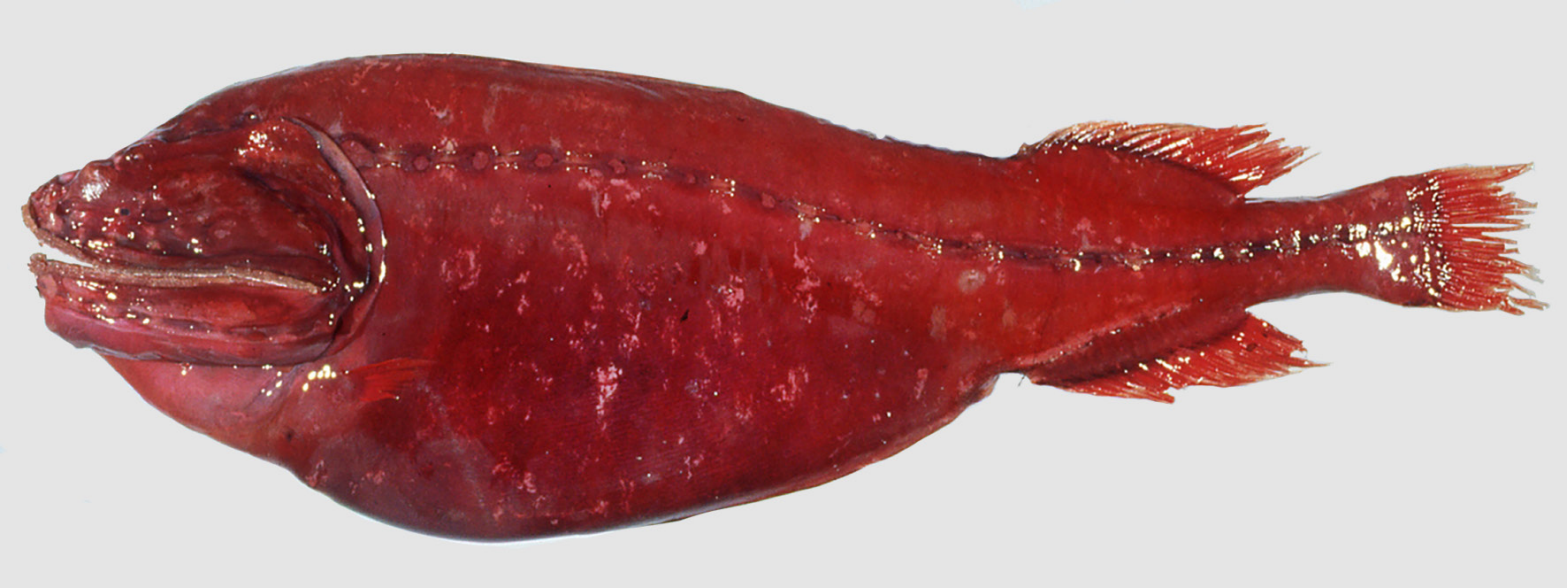
Fresh coloration of *Gyrinomimus amaokai* sp. nov., holotype, HUMZ 121996, 386 mm SL. Photo courtesy of HUMZ.

**Figure 4.**
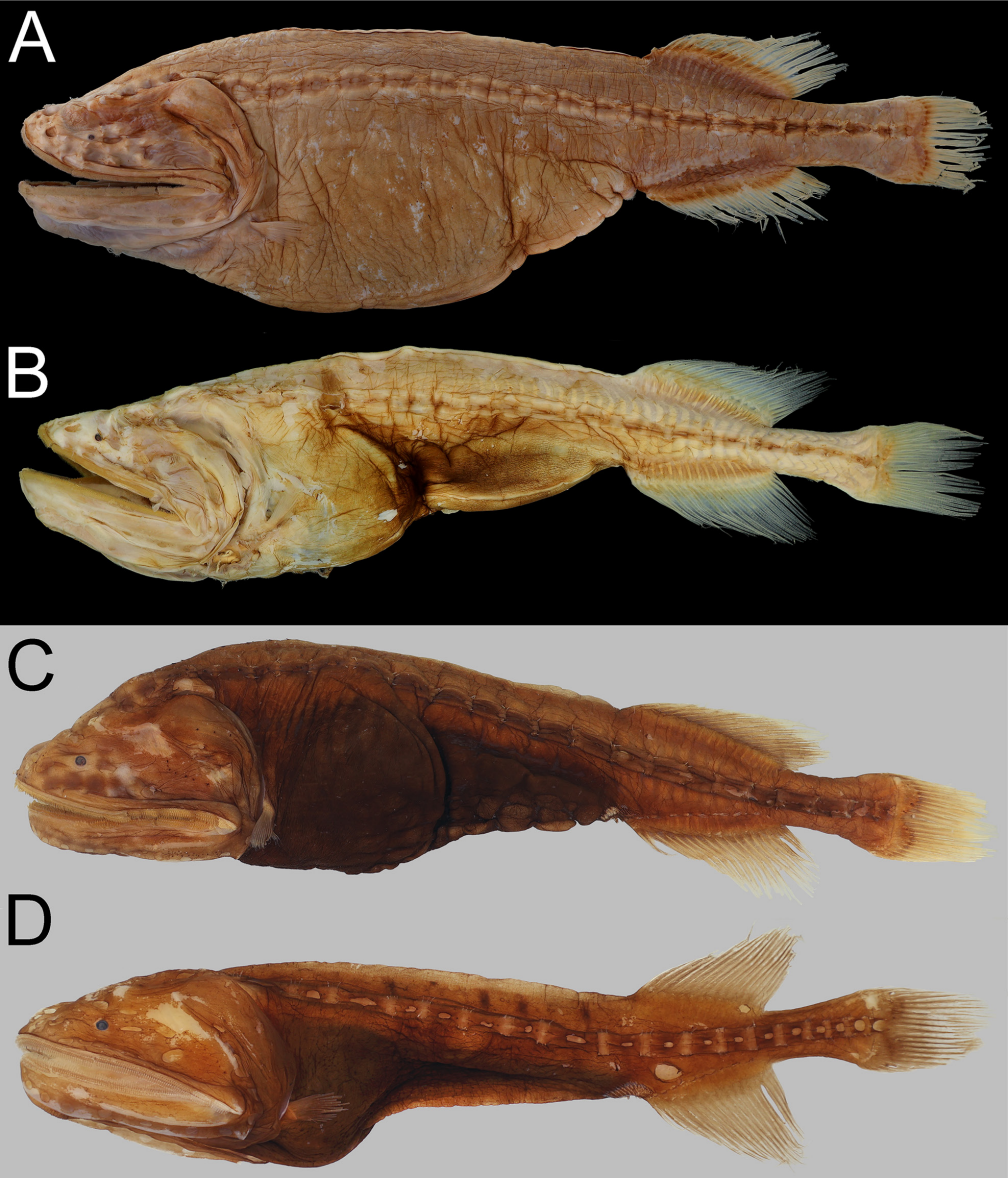
Preserved coloration of *Gyrinomimus amaokai* sp. nov. **A**. Holotype, HUMZ 121996, 386 mm SL; **B**. Paratype, NSMT-P 49412, 222.9 mm SL, left-right reversed; **C**. Paratype, SIO 60-284, 150.8 mm SL; **D**. Paratype, SIO 87-10, 89.0 mm SL. Photo by Y.-C. Hsu.

**Figure 5.**
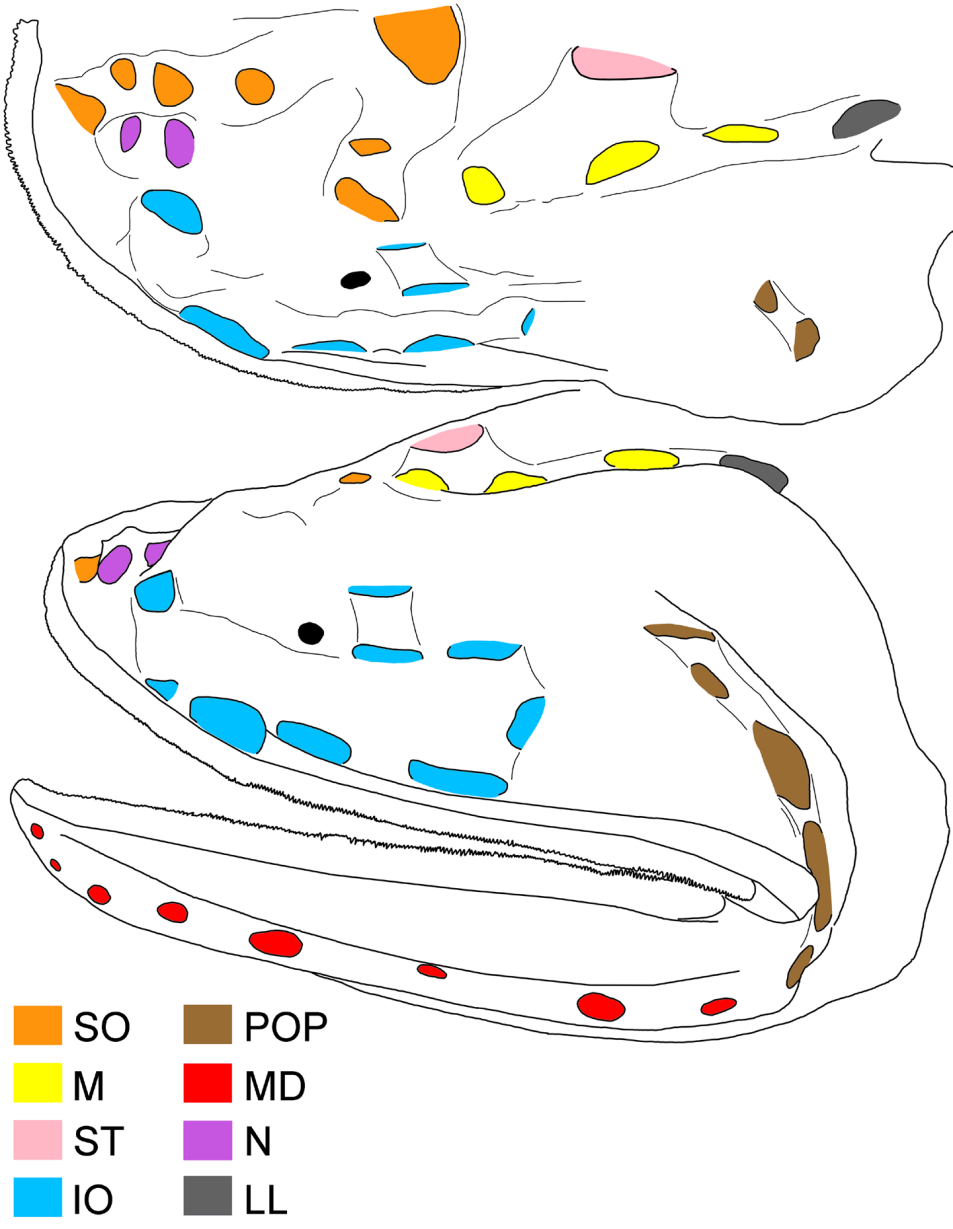
Illustration of head pores of *Gyrinomimus amaokai* sp. nov., holotype, HUMZ 121996, 386 mm SL. Upper: dorsal view; lower: lateral view. Anterior to left. Figures not to scale.

**Figure 6.**
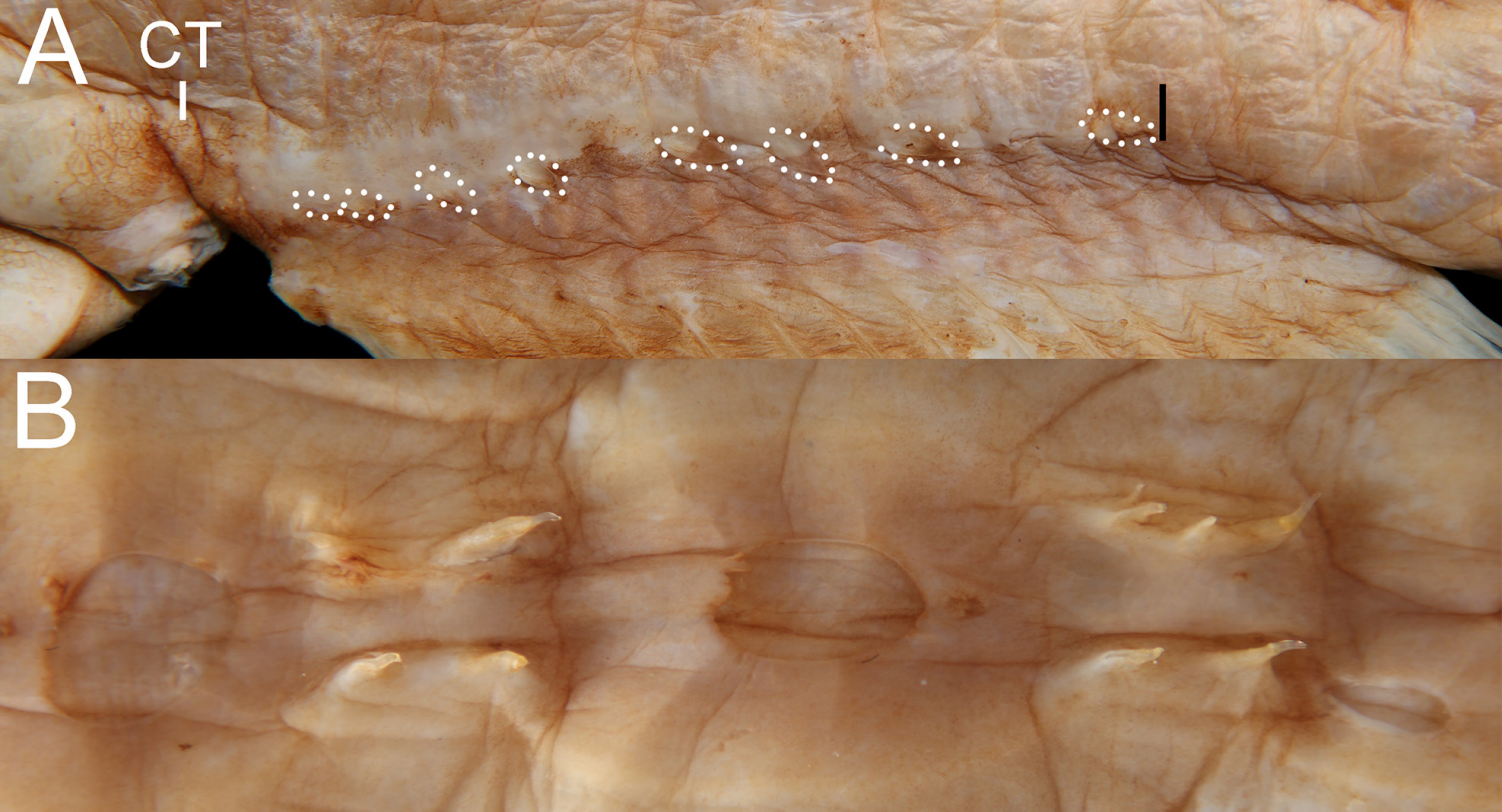
Close-up images of *Gyrinomimus amaokai* sp. nov., holotype, HUMZ 121996, 386 mm SL. **A**. Cavernous tissue (CT) around anus and above anal-fin base, with its posterior extension indicated by black bar, and anal lappets (circled); **B**. Spines on lateral-line scales. Photo by Y.-C. Hsu. Anterior to left. Figures not to scale.

**Figure 7.**
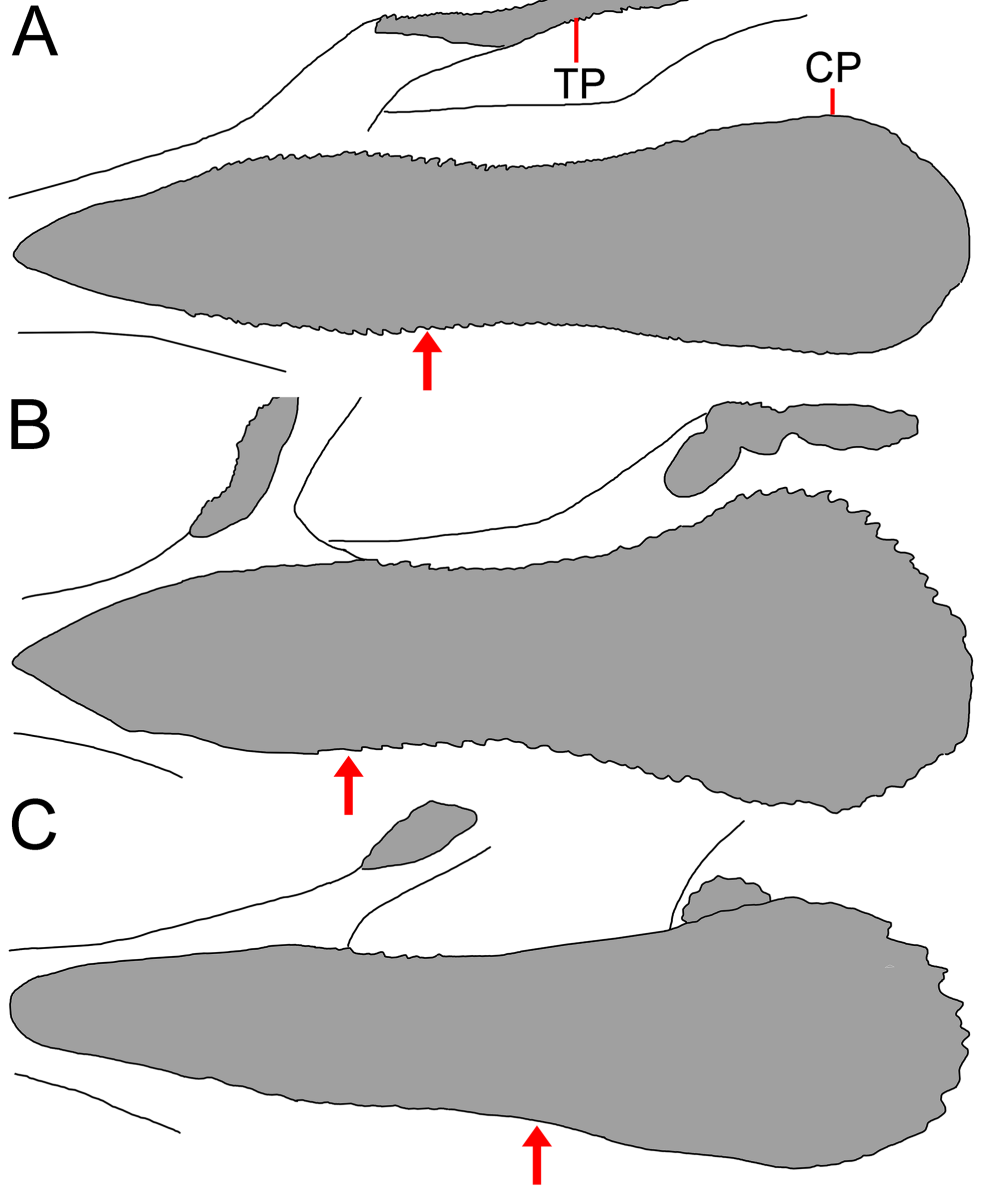
Copular tooth plates (CP) and anteriormost tooth plate (TP) on first gill arch of *Gyrinomimus amaokai* sp. nov. **A**. Holotype, HUMZ 121996, 386 mm SL; **B**. Paratype, SIO 87-10, 221.4 mm SL; **C**. Paratype, SIO 87-10, 89.0 mm SL. Arrow indicates level of eye.

Figs 3, 4, 5, 6, 7, Tables 1, 3

Gyrinomimus sp. B: Paxton 1989: 146 fig. 5C.
Gyrinomimus sp.: Amaoka 1997: 154. Aizawa in Nakabo 2002: 483 (in key). Aizawa and Doiuchi in Nakabo 2013: 576 (in key). Misawa et al. 2024: 269 (comparative material).
Cetomimus compunchus (sic, non Cetomimus compunctus Abe, Marumo & Kawaguchi, 1965): Amaoka et al. 1995: 124, fig. 195.

##### New English name.

Amaoka’s flabby whalefish.

**Table 3.** Morphometric characters of *Gyrinomimus amaokai* sp. nov., *G. johnpaxtoni* sp. nov., and *G. bruuni* Rofen, 1959. Abbreviations: A, anal-fin; D, dorsal-fin; HT, holotype; NT, non-types; P, pectoral-fin; PT, paratypes; SD, standard deviation.

	*G. amaokai* sp. nov.			*G. johnpaxtoni* sp. nov.			*G. bruuni*		
	This study			This study			This study		
	HT	PT (*n* = 6)		HT	PT (*n* = 15)		HT	NT (*n* = 6)	
		Mean (range)	SD		Mean (range)	SD		Mean (range)	SD
SL (mm)	386	89.0–365		194.2	49.8–376		83.5	85.4–192.1	
% SL									
HL	27.4	28.0 (22.9–32.7)	3.3	29.4	30.8 (26.2–36.1)	2.9	31.6	30.5 (29.3–32.3)	1.3
Head width	8.0	9.3 (8.1–10.5)	0.9	10.4	9.6 (8.1–12.0)	1.0	10.5	9.7 (8.6–10.7)	0.9
Eye diameter	0.8	0.9 (0.5–1.2)	0.3	1.1	1.4 (0.7–2.3)	0.5	1.6	1.4 (1.1–1.8)	0.3
Snout length	8.3	9.4 (7.9–10.9)	1.1	10.1	10.8 (8.7–13.2)	1.3	10.9	11.4 (8.9–12.9)	1.4
Upper-jaw length	24.1	25.6 (23.4–27.8)	1.8	25.9	27.4 (24.2–32.1)	2.3	28.6	26.9 (25.6–28.3)	1.0
Lower-jaw length	23.5	24.6 (22.8–25.5)	1.2	25.4	26.7 (22.9–30.2)	2.2	25.6	26.5 (25.5–27.8)	0.8
Posterior premaxilla to opercular margin	4.4	4.6 (3.8–6.0)	0.9	4.1	4.8 (2.5–7.5)	1.4	3.1	4.4 (3.6–5.6)	1.1
Body depth	30.5	23.0 (20.4–25.7)	1.9	26.8	22.3 (16.2–28.0)	3.6	21.8	20.8 (17.3–22.4)	2.2
Anus to anal-fin origin	1.5	1.8 (1.3–2.5)	0.4	1.6	1.5 (0.6–2.7)	0.6	1.1	1.2 (0.9–1.3)	0.2
D–A	16.5	13.8 (12.2–17.3)	1.9	17.1	12.9 (9.3–18.4)	2.6	14.7	13.4 (12.0–14.4)	1.0
Predorsal length	70.1	69.9 (64.4–72.4)	3.0	75.9	73.1 (68.4–76.1)	2.0	73.7	72.3 (68.8–75.8)	2.3
Prepectoral length	27.7	30.9 (27.0–37.1)	3.7	31.2	31.1 (24.5–35.9)	3.3	32.9	31.1 (27.6–34.0)	2.4
Preanal length	71.3	71.3 (68.9–74.1)	1.7	76.5	73.7 (69.8–77.1)	2.3	77.4	74.9 (70.9–78.6)	2.6
D base	15.7	17.1 (15.2–19.9)	1.7	18.1	16.9 (13.2–19.6)	1.7	16.5	17.3 (15.3–19.4)	1.8
A base	16.3	16.7 (14.5–17.8)	1.2	17.2	16.1 (14.0–18.4)	1.2	15.2	15.3 (13.9–18.4)	1.7
P length	7.3	6.6 (5.8–7.3)	0.6	6.2	7.2 (6.3–8.9)	0.8	9.5	7.0 (5.7–8.7)	1.5
Caudal-peduncle depth	6.3	5.8 (5.3–6.4)	0.4	6.9	5.7 (4.3–7.0)	0.7	5.5	5.2 (4.4–5.6)	0.6
Caudal-peduncle length	13.0	12.0 (11.0–12.8)	0.8	8.6	9.0 (7.1–10.8)	1.1	10.0	11.1 (9.7–12.4)	0.9
Copular tooth plate length	7.2	9.2 (8.2–10.3)	0.8	7.4	8.2 (6.9–9.3)	0.7	7.2	8.0 (7.4–8.3)	0.3
Copular tooth plate width	1.8	2.5 (2.3–2.7)	0.1	2.9	3.0 (2.4–3.7)	0.4	3.5	3.7 (3.1–4.0)	0.3
Gill filament on first arch	2.7	2.7 (2.3–3.7)	0.6	3.0	3.3 (1.6–5.5)	1.2	3.1	4.1 (2.7–6.1)	1.2
Gill filament on fourth arch	1.3	1.4 (1.1–1.6)	0.2	1.9	2.2 (1.3–3.8)	0.8	2.0	2.5 (1.6–3.3)	0.7
% Gill filament on first arch									
Gill filament on fourth arch	46.1	53.4 (43.2–64.6)	9.4	62.4	69.4 (50.0–92.1)	14.1	62.2	61.6 (44.4–75.5)	10.8
Length/width ratio of copular tooth plate	4.0	3.8 (3.3–4.1)	0.3	2.5	2.7 (2.5–3.1)	0.2	2.0	2.2 (2.0–2.4)	0.2

##### Standard Japanese name.

Oaka-kujirauo (オオアカクジラウオ).

##### Type material.

***Holotype***. • HUMZ 121996, 386 mm SL, off Shiretoko Peninsula, Hokkaido, Japan, 44°20'N, 145°7'30"E, 350–460 m, gill net, 1980–1992. ***Paratypes***. Nine specimens, 89.0–475 mm SL. • AMS I.29598-001 (formerly TH 861230), 188.4 mm SL, western Central Pacific, 12°37'12"N, 151°51'00"E, 0–2442 m, KMT net, 17 Feb. 1986. • HAF 95-01, 453 mm SL, off western coast of Iceland, northeastern Atlantic, 65°28'N, 28°00'W, 1000–1100 m, Apr.–May 1995. • HAF 2004-042, 475 mm SL, off western coast of Iceland, northeastern Atlantic, 65°25'N, 29°02'W, 1300–1370 m, 10 May 2004. • HAF 2008-025, 458 mm SL, off western coast of Iceland, northeastern Atlantic, 65°26'30"N, 28°55'36"W, 1370 m, 16 May 2008. • NSMT-P 49412 (formerly TH 840880), 222.9 mm SL, northwestern Pacific, 31°38'12"N, 157°39'36"E, 0–2320 m, R/V *Kaiyo-maru*, operation number KMT-10, station E, KMT net, 1400–1600 hrs, 1 Jun. 1984. • SIO 60-284, 150.8 mm SL, northeastern Pacific, 29°01'11.99"N, 132°09'00"W–29°11'35.99"N, 131°41'30"W, 0–3000 m, R/V *Spencer F Baird*, cruise Tethys II, station TET-31, 10-ft MWT, 13 Aug. 1963, coll. SIO party. • SIO 87-10, 2 specimens, 89.0–221.4 mm SL, northeastern Pacific, 32°50'N, 124°07'W, 0–2700 m, R/V *Melville*, cruise Atom I, station KLS 176F-3 & 5, MOCNESS net, 8 Jun. 1986, coll. K. L. Smith et al. • ZMH-ICH-0025053 (formerly ISH 1115-1979), 365 mm SL, northwestern Atlantic, 25°08'N, 67°50'W, 0–1800 m, R/V *Anton Dohrn*, station 234 IV/79, 1600 MT net, 12 Apr. 1979.

##### Diagnosis.

A species belonging to *G. bruuni* species group and differs from all congeners in having: 2–5 (very rarely 1) spines on dorsal and ventral arms of the lateral-line scales which, at least in preserved specimens, penetrate the skin of the lateral-line canal; cavernous tissue present around anus and extending to base of anterior 2^nd^–14^th^ anal-fin rays; filaments on fourth gill arch well developed, its length 43.2–64.6% filament on first arch; anal lappets 8–14, with last over anal-fin rays 12–16, and with scales; vertebrae 32–34 + 17–20 = 51–53; GRIII 1 + 1 + 1; dorsal-fin rays 17–21; anal-fin rays 17–20; lateral-line pores 18–22; lateral-line scales 17–21; IO 7 + 2 (very rarely 8 + 2) and copular tooth plate elongated, greatest width 3.3–4.1 in length.

##### Description.

Meristic and morphometric characters are provided in Tables 1, 3. Data of holotype are provided first, followed by that of paratypes in parentheses whenever available. Dorsal-fin rays 19 (17–21; *n* = 9); pectoral-fin rays 16/16 (17–22/17–21); anal-fin rays 19 (17–20; n = 9); principal caudal-fin rays 8 + 8 (7–8 + 7–8). GRI 1 + 1 + 2 (1–3 + 1 + 2–4); GRII 1 + 1 + 2 (1 + 1 + 2–4); GRIII 1 + 1 + 1 (1 + 1 + 1); gill rakers on medial face of first arch 0 (0). Lateral-line pores 21/20 (19–22/18–20); lateral-line scales 20/19 (18–22/17–19). Anal lappets 8/8 (8–14/11–14), with scales. Head pores (Fig. 5): MD 9 (8–9); POP 5; IO 7 + 2 = 9 (8 + 2 = 10 in left side of ZMH-ICH-0025053); SO 7 (7–8); M 3 (3–4); ST 1. Vertebrae 34 + 19 = 53 (32–34 + 17–20 = 51–53; *n* = 6).

Body rather slender and compressed, with abdomen slightly swollen; depth at pectoral-fin base 3.3 (3.9–4.9) in SL. Head moderate and slightly compressed, length 3.6 (3.1–4.4) in SL. Dorsum and abdomen with distinct central ridge. Nostrils similar to head lateral-line pores, closer to tip of snout than eye; anterior nostril slightly smaller than posterior one; both nostrils oval; nasal organ mostly in anterior nostril. Eye tiny, length 36.2 (24.6–49.3) in HL. Interorbital broadly concave, with distinct concavity just on top of head at level of eye in holotype, and no distinct concavity in paratypes; anterolateral projection of frontal slightly posterior to eye. Caudal peduncle short and deep, length and depth 2.1 (1.8–2.7) and 4.3 (4.3–5.8) in HL, respectively. Cavernous tissue surrounding anus, and extending to base of anal-fin ray 14 (2–7; Fig. 6A); present on dorsal-fin origin of holotype and one of SIO 87-10, 221.4 mm SL; present on mouth roof, esophagus, and body wall behind fourth gill arch. Anal lappets well developed (Fig. 6A), along anal-fin base to base of anal-fin ray 16 (12–16); scales present on anal lappets.

Mouth enormous and oblique; upper-jaw length 1.1 (1.0–1.2) in HL; lower jaw with two posterior spines, with ventral spine blunt and lateral spine short, blunt, and covered under skin. Jaw teeth elongated, in distinct longitudinal rows; inner row of teeth largest; premaxillary and dentary symphyses edentate. Upper jaw with 8 (3–6) rows of teeth anteriorly, its number slightly decreases posteriorly, with 6 (2–5) rows posteriorly. Lower-jaw teeth similar to those of upper jaw, with 9 (4–8) rows anteriorly and 6 (2–5) rows posteriorly. Vomerine, palatine, and ectopterygoid teeth similar in shape to jaw teeth. Vomerine tooth patch domed or slightly rounded, with 7 (4–7) rows of teeth. Palatine tooth patch beginning at level of middle of vomer, with 11 (2–7) rows of teeth; its posterior end anterior to level of eye. Ectopterygoid tooth patch narrow, with ~ 18 (4–16) teeth rows and nearly attached to palatine tooth patch (ectopterygoid and palatine tooth patches fused in ZMH-ICH-0025053); its origin anterior to posterior end of palatine, extending posteriorly beyond rictus and ending anterior to end of premaxilla. Copular tooth patch (Fig. 5) elongated, its anterior margin slightly pointed, slightly concave at middle, and slightly widens posteriorly; its length and width 3.8 (2.5–3.3) and 15.3 (10.0–13.1) in HL, respectively; length/width ratio 4.0 (3.3–4.1).

Free gill arches 3, with small slit behind ventral arm of third arch near angle, extending ~ 1/2 (1/2–2/3) length of filaments-bearing part of ventral arm; dorsal arm of arch I 2/3 (2/3–4/5) free; dorsal arm of arch II 1/2 (1/2–2/3) free. Gill filaments of all arches moderately long and well developed; length of filaments on arch IV 46.1 (43.2–64.6)% length of filaments on arch I. Gill rakers forming plates on lateral face of outer three arches; none on medial face of all arches. Anterior margin of anteriormost hypobranchial tooth plate of first arch posterior to anterior margin of copular tooth plate. Two dorsal pharyngeal tooth plates, both domed or oval; ventral pharyngeal tooth plate absent.

Lateral-line pores large, anterior pores almost as wide as canal, becoming smaller posteriorly, to ~ 1/4 or 1/5 of canal length above anal fin, and slightly widens posteriorly. Lateral line without flaps anterior to pores on posterior portion; keels absent to moderately developed. Two or three papillae on anterior and 1 or 2 on posterior margins of pores. Vertical row of ~ 4 (5–8) papillae on base of caudal fin. Dorsal accessory line of neuromasts beginning slightly before level of upper limit of gill opening, with 20 (23–35) papillae scattered on dorsum and onto mid-dorsal keel. Lateral-line scales with 1–5 (modally 3–4; very rarely 1) spines on dorsal and ventral arms and penetrate skin of lateral-line canal (Fig. 6B).

Lateral-line system of head well developed. Head with numerous scattered papillate superficial neuromasts, 5–25 (damaged in holotype) papillae on tip of snout between midline and first supraorbital pore; no papillae above anterior nostril; 0 (0–6) papillae posteroventral to anterior nostril; 2 (2–3) papillae dorsal to first infraorbital pore; 2 (0–2) papillae dorsal to second and third infraorbital pores; 8 (0–16) papillae dorsal to first main-canal pore; 2 (1–2) papillae medial to supratemporal pore; 3 (6–12) papillae anterior to preopercular pores; 4 (4–6) papillae on opercle; ~ 10–30 (damaged in holotype) papillae scattered above mandibular pores, becoming denser posteriorly.

Dorsal and anal fins far back on body, with dorsal-fin origin at same (or slightly anterior or posterior to) vertical through that of anal fin; their tips pointed posteriorly. Dorsal fin with ~ 10^th^–16^th^ (~ 7^th^–18^th^; *n* = 2) rays branched, others unbranched. Anal fin with ~ 8^th^–16^th^ (8^th^–19^th^; *n* = 4) rays branched, others unbranched. Dorsal- and anal-fin rays branched at tip, and becoming proximally as growth. Pectoral fin low, broad-based, directed posterodorsally, and small, length 3.7 (3.4–4.3) in HL; middle rays longest; all rays simple. Subpectoral organ only discernable in faded specimens.

##### Coloration.

When fresh (Fig. 3), head, body, and all fins uniformly bright red. When preserved (Fig. 4), body brown to pale. Gill arches, rakers and filaments, and inner face of operculum pale. Teeth transparent. Peritoneum and stomach black. Esophagus and mouth roof with dark marble patterns. Abdomen with dark reticulated pattern.

##### Etymology.

We are pleased to name this species after Dr. Kunio Amaoka, Professor Emeritus of Hokkaido University, for his great contributions to our knowledge of deep-sea fishes, documenting the fresh condition of holotype, and his long-term friendship with the authors.

##### Distribution.

Currently known from specimens collected from the Pacific and Atlantic Oceans in the Northern Hemisphere between 11°N and 66°N.

##### Remarks.

The subpectoral organ is a dark structure situated anteroventrally to the pectoral-fin base (Paxton 1989). It is visible from the faded specimens (holotype, NSMT-P 49412, and ZMH-ICH-0025053; Figs 3, 4A, B) as a dark-brown spot below and anterior to pectoral-fin base. However, we are unable to confirm this structure in other specimens of which the dark pigments mostly remain on body.

#### Gyrinomimus johnpaxtoni

Taxon classification

Animalia

CetomimiformesCetomimidae

Su & Ho sp. nov.

3C484A33-8D19-5843-9CD1-103DF7861BF9

https://zoobank.org/94EFE6B5-ADB3-4E53-B85F-89EBD898CEE0

**Figure 8.**
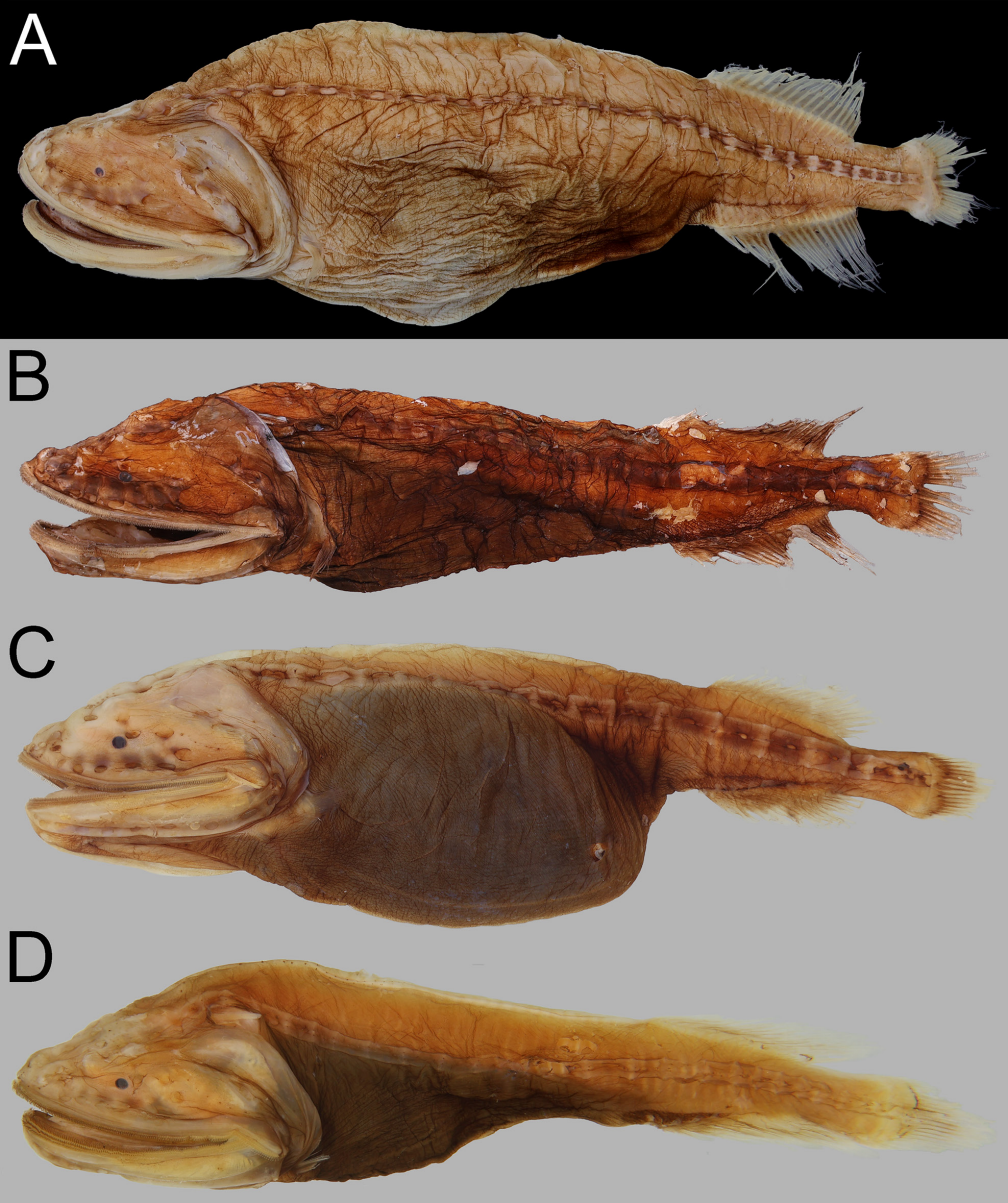
Preserved coloration of *Gyrinomimus johnpaxtoni* sp. nov. **A**. Holotype, LACM 36034-7, 194.2 mm SL; **B**. Paratype, ASIZP 60128, 158.8 mm SL; **C**. Paratype, SIO 70-302, 116.9 mm SL; **D**. Paratype, LACM 31109-1, 94.5 mm SL. Photo by Y.-C. Hsu.

**Figure 9.**
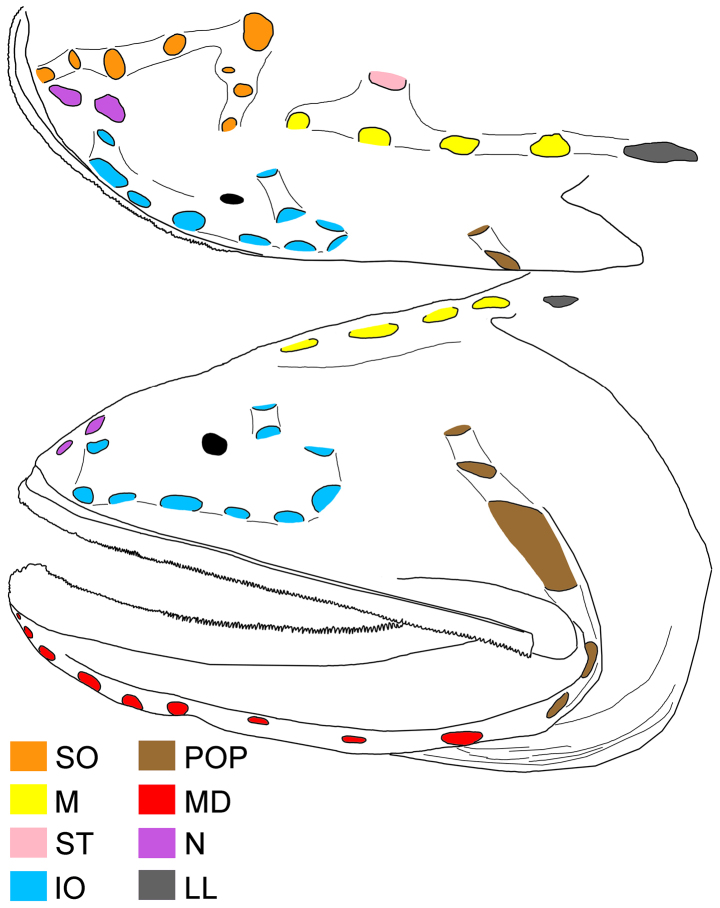
Illustration of head pores of *Gyrinomimus johnpaxtoni* sp. nov., holotype, LACM 36034-7, 194.2 mm SL. Upper: dorsal view; lower: lateral view. Anterior to left. Figures not to scale.

**Figure 10.**
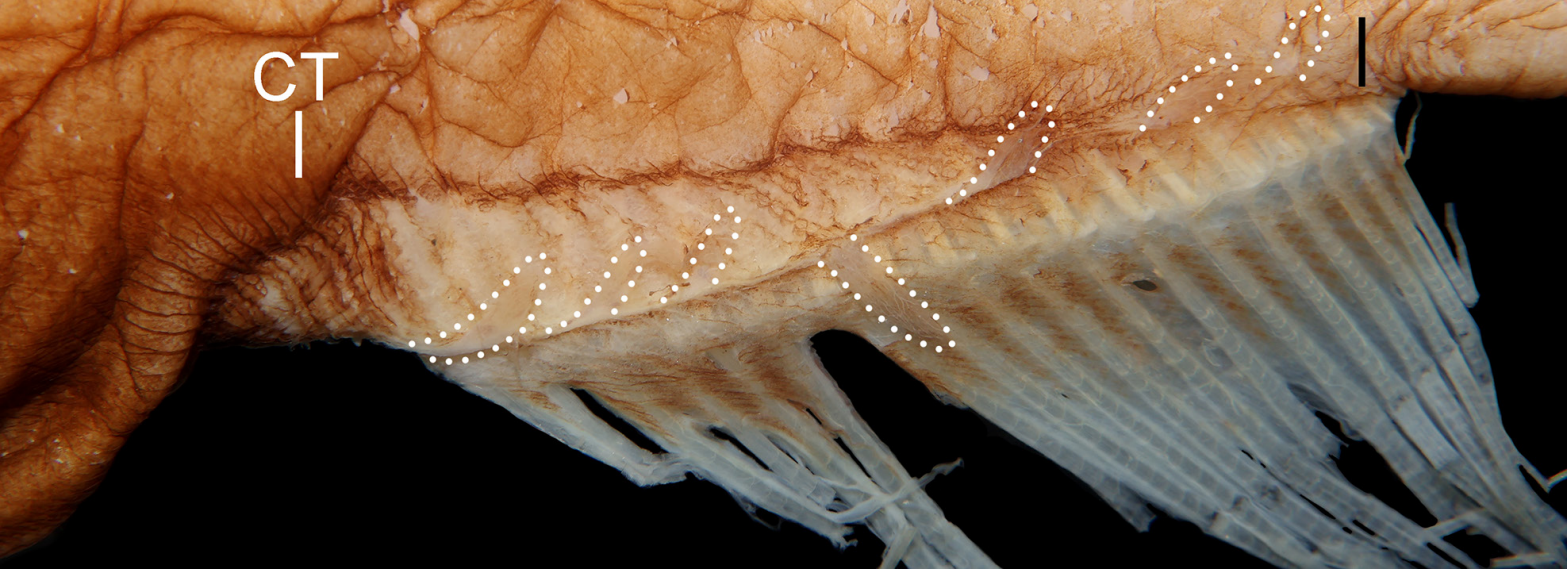
Close-up images of *Gyrinomimus johnpaxtoni* sp. nov., holotype, LACM 36034-7, 194.2 mm SL, featuring cavernous tissue (CT) around anus and above anal-fin base, with its posterior extension indicated by black bar, and anal lappets (circled). Photo by Y.-C. Hsu. Anterior to left. Figure not to scale.

**Figure 11.**
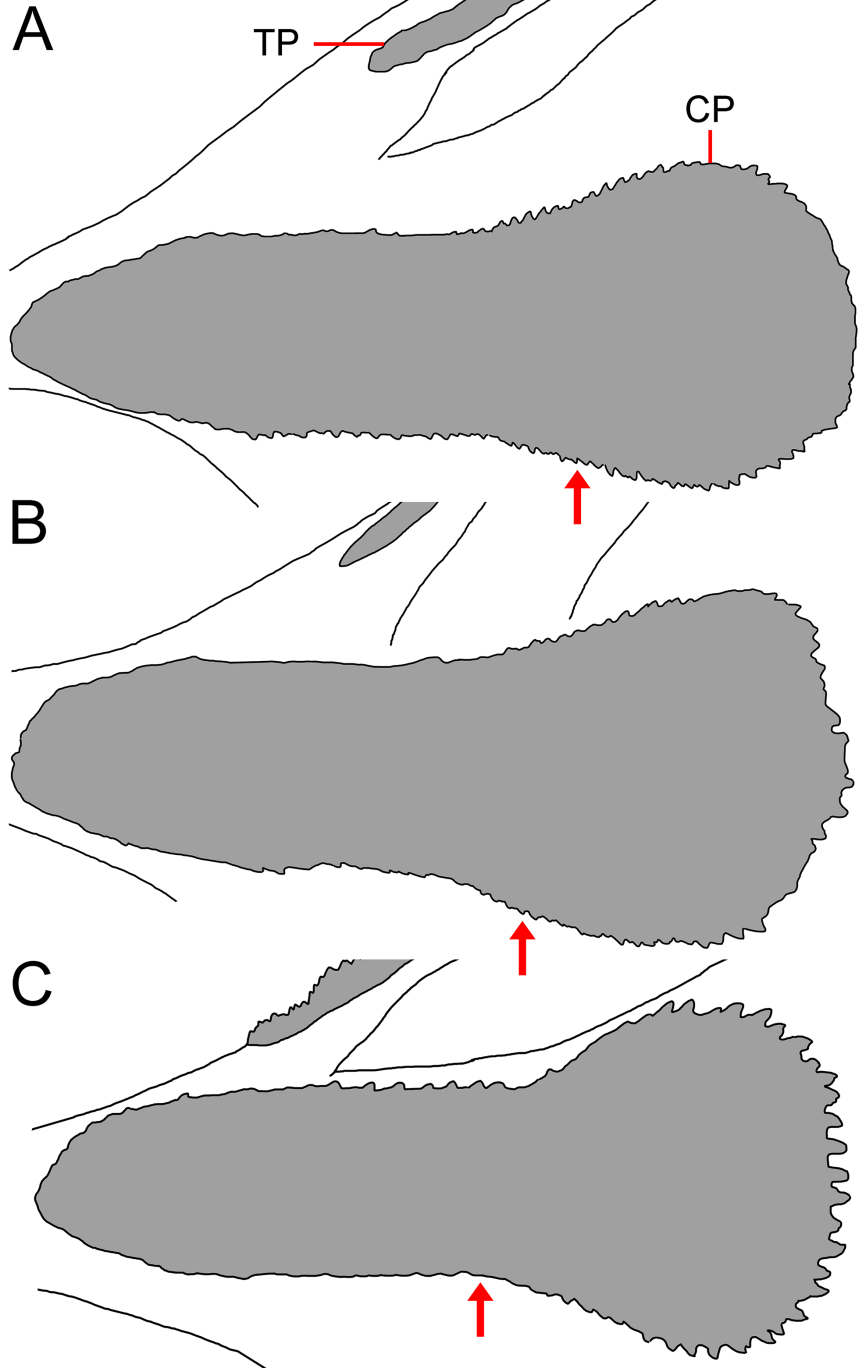
Copular tooth plates (CP) and anteriormost tooth plate (TP) on first gill arch of *Gyrinomimus johnpaxtoni* sp. nov. **A**. Paratype, LACM 6903-21, 263.8 mm SL; **B**. Holotype, LACM 36034-7, 194.2 mm SL; **C**. Paratype, LACM 31109-1, 94.5 mm SL. Arrow indicates level of eye. Anterior to left. Figures not to scale.

Figs 8, 9, 10, 11, Tables 1, 3

Gyrinomimus sp. C: Paxton 1989: 144, 176, figs 4e, 26.
Gyrinomimus bruuni (non Rofen): Misawa et al. 2024: 274, figs 4, 5 (description, Japan).

##### New English name.

Paxton’s flabby whalefish.

##### New Chinese name.

帕氏小圓鯨鯛.

##### Standard Japanese name.

Hitaguro-kujirauo (ヒタグロクジラウオ).

##### Type material.

***Holotype***. • LACM 36034-7, 194.2 mm SL, off Indonesia, Banda Sea, western Pacific, 4°57'30"S,130°11'42"E, R/V *Alpha helix*, 28 Apr. 1975. ***Paratypes***. Fifteen specimens, 65.0–376 mm SL. • AMS I.29642-001(formerly TH 861050), 176.7 mm SL, northwestern Pacific, 31°49'N, 154°47'E, 0–2559 m, R/V *Kaiyo-maru*, 0630–1101 hrs, 1 Jun. 1984. • ASIZP 60128, 158.8 mm SL, off Daxi fishing port (ca 24°53'37"N, 121°55'26"E), Yilan, northeastern Taiwan, northwestern Pacific, 27 Jan. 1999, coll. P.-L. Lin. • HUMZ 234625, 65.1 mm SL, off Ibaraki Prefecture, Japan, 36°31'11"N, 141°21'29.2"E–36°31'27.5"N, 141°21'55.0"E, 886–892 m, R/V *Wakataka-maru*, station H900, otter trawl, 27 October 2023, coll. K. Fujiwara, S. Tokioka, and M. Furusho; Genbank accession number: LC799418. • LACM 6903-21, 263.8 mm SL, off San Clemente Island, California, USA, northeastern Pacific, 32°16'00"N, 117°43'20"W, R/V *Velero*, 21 Feb. 1966. • LACM 30037-11, 2 specimens, 66.0–97.0 mm SL, 26.7 miles from San Juanito Island, Mexico, Gulf of California, eastern Pacific, 21°39'30"N, 106°58'00"W, R/V *Velero IV*, 11 Nov. 1967. • LACM 31104-2, 65.0 mm SL, off Mexico, Middle American Trench, northeastern Pacific, 16°17'00"N, 100°14'00"W, R/V *Velero IV*, 13 Jan. 1970. • LACM 31109-1, 94.5 mm SL, off Mexico, Middle American Trench, northeastern Pacific, 17°47'00"N, 103°20'30"W, R/V *Velero IV*, 15 Jan. 1970. • NSMT-P 49414 (formerly TH 861662), 376 mm SL, western Pacific, 12°35'N, 151°52'E, 0–1903 m, R/V *Kaiyo-maru*, operation number KMT-13, station M., KMT net, 0620–0930 hrs, 16 Feb. 1986. • SIO 59-201, 166.6 mm SL, Carmen Basin, Mexico, Gulf of California, northeastern Pacific, 26°15'N, 110°36'W, 0–930 fathoms (=1700 m), R/V *Horizon*, cruise Vermilion Sea, station VS 120-18 trawl #3, 10-ft IKMT net, 21 Mar. 1959, coll. R.L. Wisner & SIO party. • SIO 70-302, 116.9 mm SL, Kamchatka Trench, Bering Sea, northeastern Pacific, 52°25'53.99"N, 161°37'12"W–52°15'11.99"N, 161°26'48"W, 0–2500 m, R/V *Melville*, cruise Antipode III, 10-ft IKMT net, 3 Aug. 1970, coll. D. C. Dockins & SIO party. • ZIN 47867, 169.0 mm SL, western Pacific, 15°37'N, 152°02'E, no other data. • ZMH-ICH-0110459 (formerly ISH 1573-1971), 209.7 mm SL, off west Namibia, southern Atlantic, 27°14'S, 2°56'E, 1900–2000 m, 1 Apr. 1971. • ZMH-ICH-0111307 (formerly ISH 87-1973), 276.2 mm SL, north Atlantic, 45°18'N, 48°08'W, 1720–1725 m, FRV *Walther Herwig II*, station 272/73, 13 May 1973, coll. Bohl. • ZMH-ICH-0118202 (formerly ISH 1495-1979), 49.8 mm SL, 28°41'N, 60°54'W, ca 2000 m, R/V *Anton Dohrn 2*, station 2376/79, 1600 MT net, 20 Apr. 1979.

##### Non-types.

Five specimens, 64.8–339 mm SL. • LACM 30530-30, 253.8 mm SL, off San Clemente Island, California, USA, northeastern Pacific, 32°19'45.0"N, 117°49'07.0"W, R/V *Velero IV*, 27 Jan. 1986. • NSMT-P 71816 (formerly TH 831159), 58.7 mm SL, north Pacific, 23°17'N, 149°51'E, no other data. • MNHN 1992-0325, 64.8 mm SL, off New Caledonia, 22°12’S, 165°48’E, 640 m, 29 Apr. 1971. • SIO 20-23, 339 mm SL, USA, northeastern Pacific, 31°41'04"N, 121°15'00"W–31°44'27"N, 121°18'15"W, 2000–2499 m, R/V *Sally Ride*, cruise SR2007 SiphWeb, station 4, 10 m MOCNESS net, 26–27 Aug. 2020, coll. A. Choy et al. • SIO 51-375, 69.8 mm SL, north Pacific, 31°54'17.99"N, 152°21'36"W–31°36'30"N, 152°03'36"W, 0–1700 fathoms (= 3109 m), R/V *Horizon*, cruise Northern Holiday, station NH-25,10-ft MWT net, 15 Sep. 1951, coll. R.L. Wisner and SIO. • SIO 61-47, 75.9 mm SL, south Pacific, 25°52'S, 155°44'W–25°40'S, 155°34'W, 0–2550 m, R/V *Argo*, cruise Monsoon Exp, station VII-24 trawl 19, 10-ft IKMT net, 15 Sep. 1951, coll. C. Russell & J. Coatsworth.

##### Diagnosis.

A species belonging to *G. bruuni* species group and differs from all congeners in having: no distinct spines on the dorsal and ventral arms of the lateral-line scales that protrude from skin; cavernous tissue present around anus and extending to base of anal-fin rays 6–21; filaments on fourth gill arch well developed, its length 50.0–92.1% filament on first arch; anal lappets 4–9 with the last over base of anal-fin ray 8–15 and with scales; vertebrae 35–38 + 18–20 = 54–58; GRIII 0 + 1 + 1; dorsal-fin rays 19–21; anal-fin rays 18–21; lateral-line pores 19–25; lateral-line scales 18–24; and copular tooth plate moderately long and broad, greatest width 2.5–3.1 in length.

##### Description.

Meristic and morphometric characters are provided in Tables 1, 3. Data of holotype are provided first, followed by that of paratypes in parentheses whenever available. Dorsal-fin rays 19 (19–21); pectoral-fin rays 16/16 (16–21/15–19); anal-fin rays 19 (18–21); principal caudal-fin rays 8 + 8 (7–8 + 7–8). GRI 1 + 1 + 2 (1 + 1 + 2–3); GRII 1 + 1 + 2 (0–1 + 1 + 2–4); GRIII 0 + 1 + 1 (0 + 1 + 1); medial GRI 0 (0). Lateral-line pores 23/22 (19–25/19–22); lateral-line scales 20/19 (18–24/18–21). Anal lappets 7/7 (4–9/4–8), with scales. Head pores (Fig. 8): MD 9 (8–11); POP 5 (3–5); IO 8 + 2 = 10; SO 8 (7–8); M 4 (3–4); ST 1. Vertebrae 38 + 18 = 56 (35–38 + 18–20 = 54–58; *n* = 14).

Body rather slender and compressed, depth at pectoral-fin base 3.7 (3.6–6.2) in SL. Head moderate and slightly compressed, length 3.4 (2.8–3.8) in SL. Dorsum and abdomen with distinct central ridge. Nostrils similar to head lateral-line pores, closer to tip of snout than eye; anterior nostril slightly smaller than posterior one; both nostrils oval; nasal organ mostly in anterior nostril. Eye tiny, diameter 27.2 (12.5–37.9) in HL. Interorbital broadly concave, without distinct pits (convex in smaller specimens); anterolateral projection of frontal above or posterior to eye. Caudal peduncle rather short and deep, length and depth 3.4 (2.8–4.7) and 4.2 (4.1–6.5) in HL, respectively. Cavernous tissue (Fig. 10) surrounding anus and extending to base of last ana-fin ray (base of 6^th^ to last anal-fin ray), and sometimes onto anal lappets; present on mouth roof, esophagus, and body wall behind fourth gill arch. Anal lappets well developed (Fig. 10), along anal-fin base, last above base of anal-fin ray 16 (7–15); scales present on anal lappets.

Mouth enormous and oblique; upper-jaw length 1.1 (1.1–1.2) in HL; lower jaw with two posterior spines, with ventral spine blunt and lateral spine rather stout, double tipped, and covered under skin. Jaw teeth elongated, in distinct longitudinal rows; inner row of teeth largest; premaxillary and dentary symphyses edentate. Upper jaw with 7 (3–7) rows of teeth anteriorly, its number slightly decreases posteriorly or number unchanged, with 5 (2–6) rows posteriorly. Lower-jaw teeth similar to those of upper jaw, with 10 (3–9) rows anteriorly and 7 (2–7) rows posteriorly. Vomerine, palatine, and ectopterygoid teeth similar in shape to jaw teeth. Vomerine tooth patch domed, with 8 (2–10) rows of teeth. Palatine tooth patch beginning at level of middle of vomer, with 7 (2–11) rows of teeth; its anterior end anterior to level of eye. Ectopterygoid tooth patch broad, with ~ 13 (2–18) teeth rows and nearly attached to palatine tooth patch; its origin anterior to posterior end of palatine, extending posteriorly beyond rictus and before end of premaxilla. Copular tooth patch (Fig. 9C) teardrop shaped, rather oval in smaller specimens; its anterior margin rounded or slightly pointed, with slightly concave at middle and slightly widens posteriorly; its length and width 4.0 (3.2–5.2) and 10.0 (8.6–14.6) in HL, respectively; length/width ratio 2.5 (2.5–3.1).

Free gill arches 3, with rather long slit behind ventral arm of third arch near angle, extending approx. entire length (~ 60% to entire) of filaments-bearing part of ventral arm; dorsal arm of arch I 1/3 (1/3–1/2) free; dorsal arm of arch II 1/3 (1/3–1/2) free. Gill filaments of all arches moderately long and well developed; length of filaments on arch IV 62.4 (50.0–92.1)% length of filaments on arch I. Gill rakers forming plates on lateral face of outer three arches; none on medial face of all arches. Anterior margin of anteriormost hypobranchial tooth plate of first arch posterior to anterior margin of copular tooth plate. Two dorsal pharyngeal tooth plates, both domed and slightly oval; no ventral pharyngeal tooth plate.

Lateral-line pores large, anterior pores almost as wide as canal, becoming smaller posteriorly, to ~ 1/4 or 1/5 of canal length. Lateral line with small flap anterior to pores on posterior portion (two specimens without flaps); keels present between pores on posterior portion of body (four specimens without keels). Zero to two papillae on both anterior and posterior margins of pores. Vertical row of ~ 3–6 (damaged in holotype) papillae on base of caudal fin. Dorsal accessory line of neuromasts beginning slightly before level of upper limit of gill opening, with 16 (17–38) papillae scattered on dorsum and onto mid-dorsal keel. Lateral-line scales with none or one spine on dorsal and ventral arms of the lateral-line scales and not penetrate skin of lateral-line canal.

Lateral-line system of head well developed. Head with numerous scattered papillate superficial neuromasts, 16 (11–22) papillae on tip of snout between midline and first supraorbital pore; 0 (0–1) papillae above anterior nostril; 4 (3–6) papillae posteroventral to anterior nostril; 4 (1–7) papillae dorsal to first infraorbital pore; 1 (0–1) papillae dorsal to second and third infraorbital pores; 17 (13– 26) papillae dorsal to first main-canal pore; 1 (0–1) papillae medial to supratemporal pore; 9 (4–9) papillae anterior to preopercular pores; 1–5 (damaged in holotype) papillae on opercle; ~ 9–18 (damaged in holotype) papillae scattered above mandibular pores, becoming denser posteriorly.

Dorsal and anal fins far back on body, with dorsal-fin origin slightly anterior to that of anal fin; their tips pointed posteriorly. Dorsal fin with ~ 9^th^–17^th^ (*n* = 3; fin rays damaged in most specimens) rays branched, others unbranched. Anal fin with middle ~ 8^th^–18^th^ (*n* = 3; fin rays damaged in most specimens) rays branched, others unbranched. Dorsal- and anal-fin rays branched at tip, and becoming proximally as growth. Pectoral fin low, broad-based, directed posterodorsally, and rather short, length 4.7 (3.4–5.7) in HL; middle rays longest; all rays simple. Subpectoral organ only discernable in SIO 70-302, LACM 31104-2, and 1 of LACM 30037-11, 97.0 mm SL.

##### Coloration.

When fresh (Misawa et al. 2024, fig. 4), body and all fins uniformly black, with exposed fin rays and teeth transparent. When preserved (Fig. 7), body brown to pale. Gill arches, rakers and filaments, and inner face of operculum pale. Teeth transparent. Peritoneum and stomach black. Esophagus and mouth roof with dark marble patterns. Abdomen with dark reticulated pattern.

##### Distribution.

Currently known from specimens collected in the Pacific and Atlantic Oceans between 26°S and 53°N.

##### Etymology.

We are pleased to name this species after the late Dr. John R. Paxton, Australian Museum, for his great contributions to our knowledge of cetomimids, and establishing the foundation of this study.

#### Gyrinomimus johnsoni sp. nov.

Taxon classification

Animalia

CetomimiformesCetomimidae

56537627-414C-5505-BEAE-538BBCC76ACC

https://zoobank.org/10218CDF-D8D6-482F-891F-EA44CE6E479E

**Figure 12.**
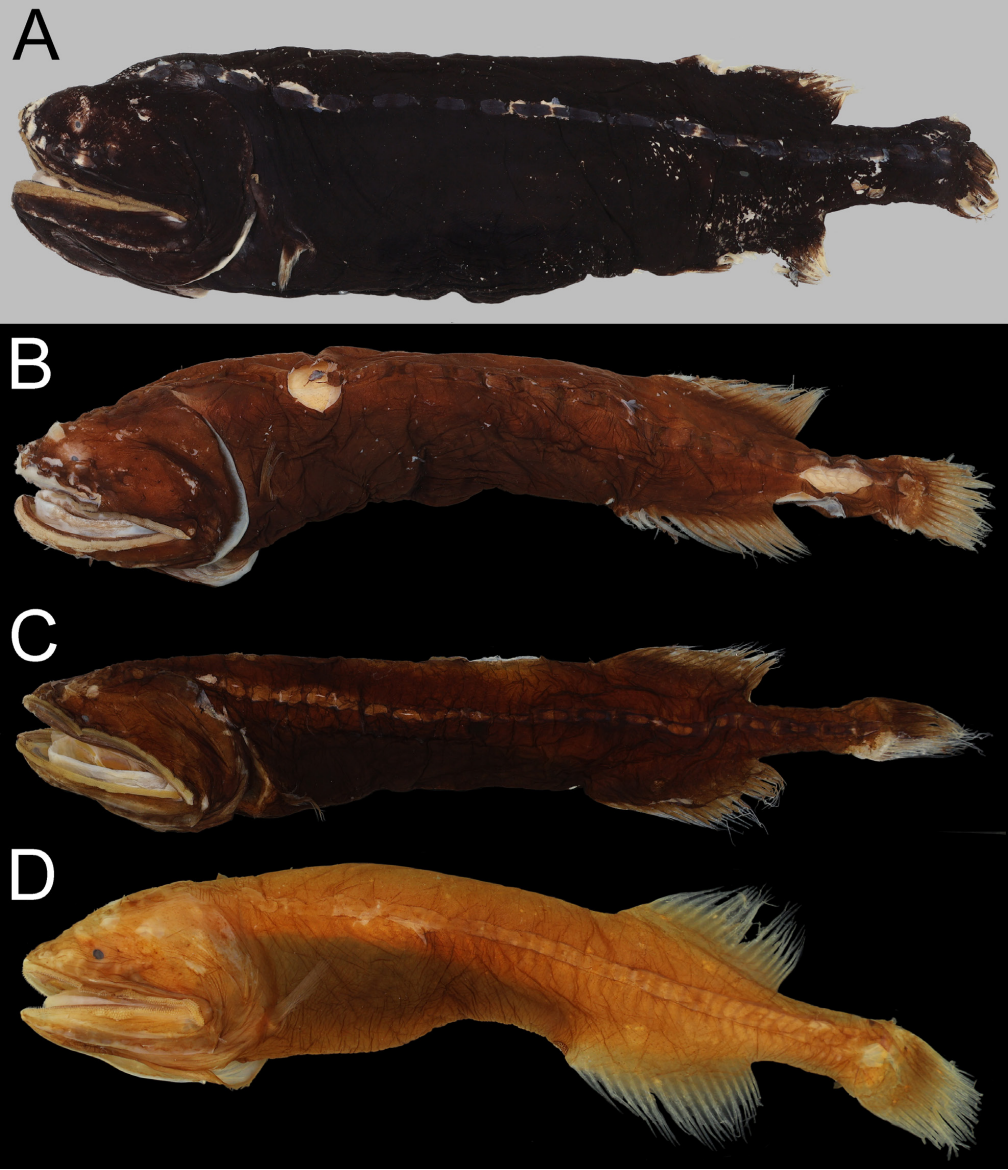
Preserved coloration of *Gyrinomimus johnsoni* sp. nov. **A**. Paratype, USNM 296934, 290.0 mm SL; **B**. Holotype, USNM 283095, 194.0 mm SL; **C**. Non-type, AMS I.29640-001, 147.2 mm SL; **D**. Paratype, SIO 70-306, 95.0 mm SL. Photo by Y.-C. Hsu.

**Figure 13.**
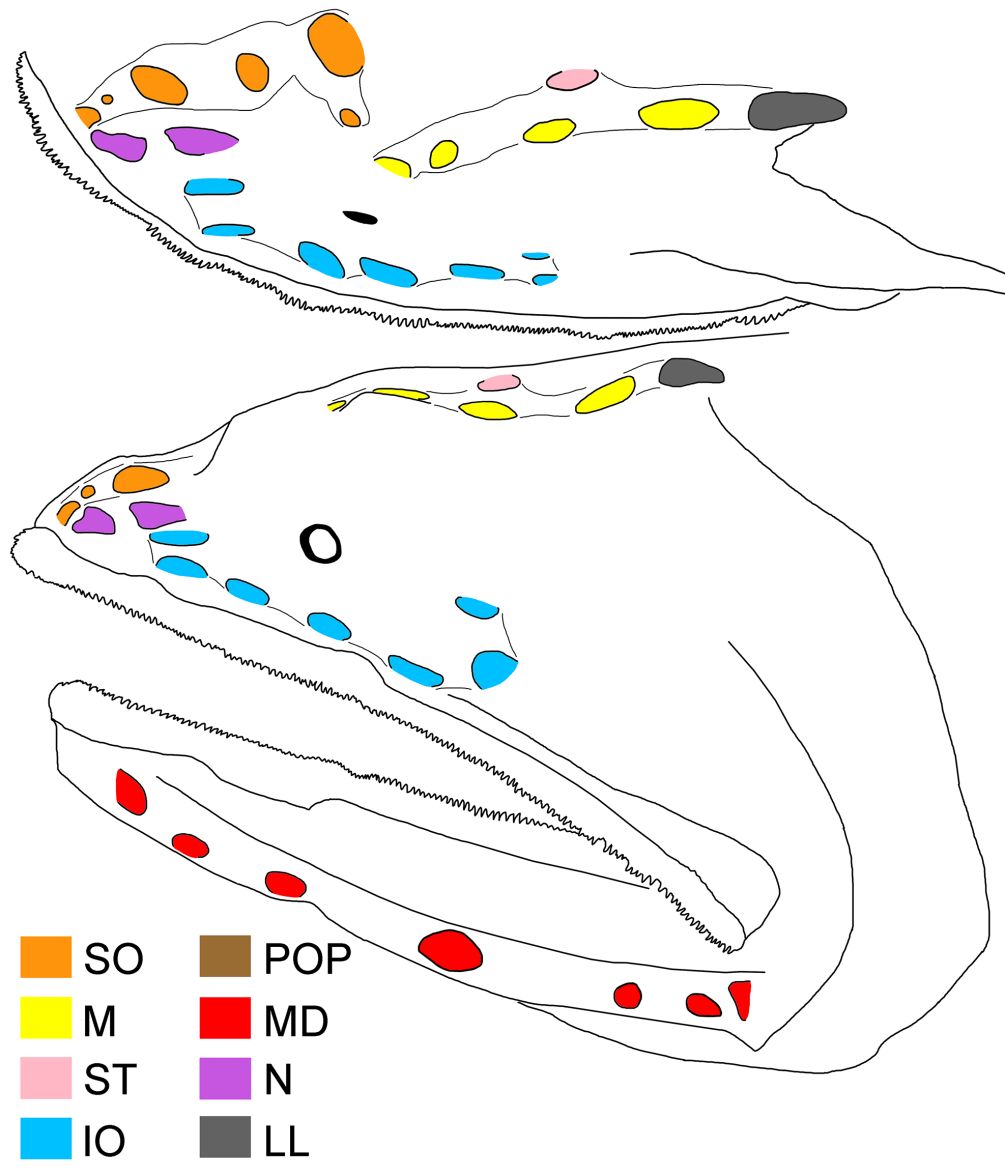
Illustration of head pores of *Gyrinomimus johnsoni* sp. nov., paratype, NSMT-P 71817, 109.1 mm SL. Upper: dorsal view; lower: lateral view. Anterior to left. Figures not to scale.

**Figure 14.**
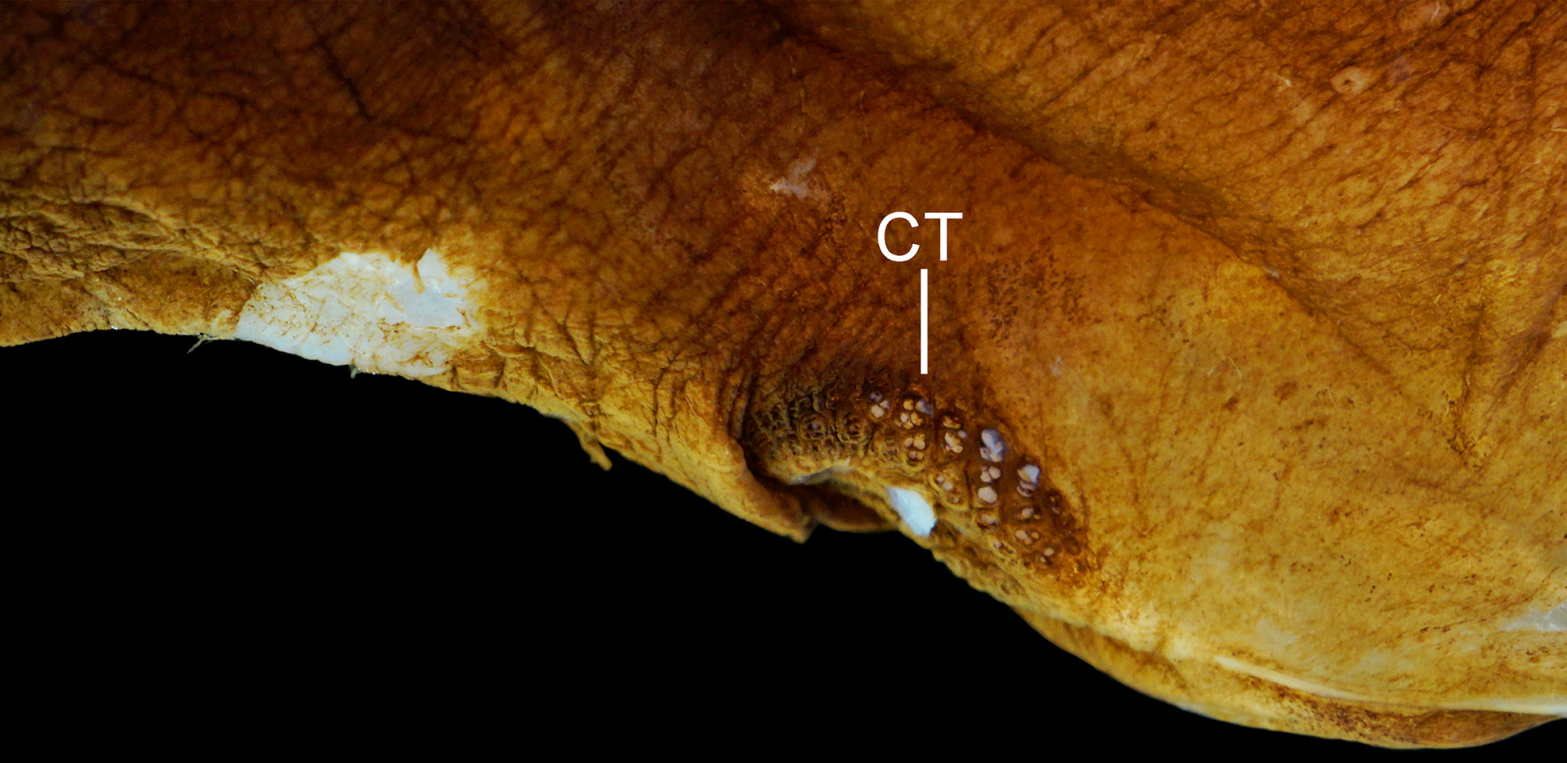
Close-up images of *Gyrinomimus johnsoni* sp. nov., paratype, NSMT-P 71817, 109.1 mm SL, featuring cavernous tissue around anus. Photo by Y.-C. Fan. Anterior to left. Figure not to scale.

**Figure 15.**
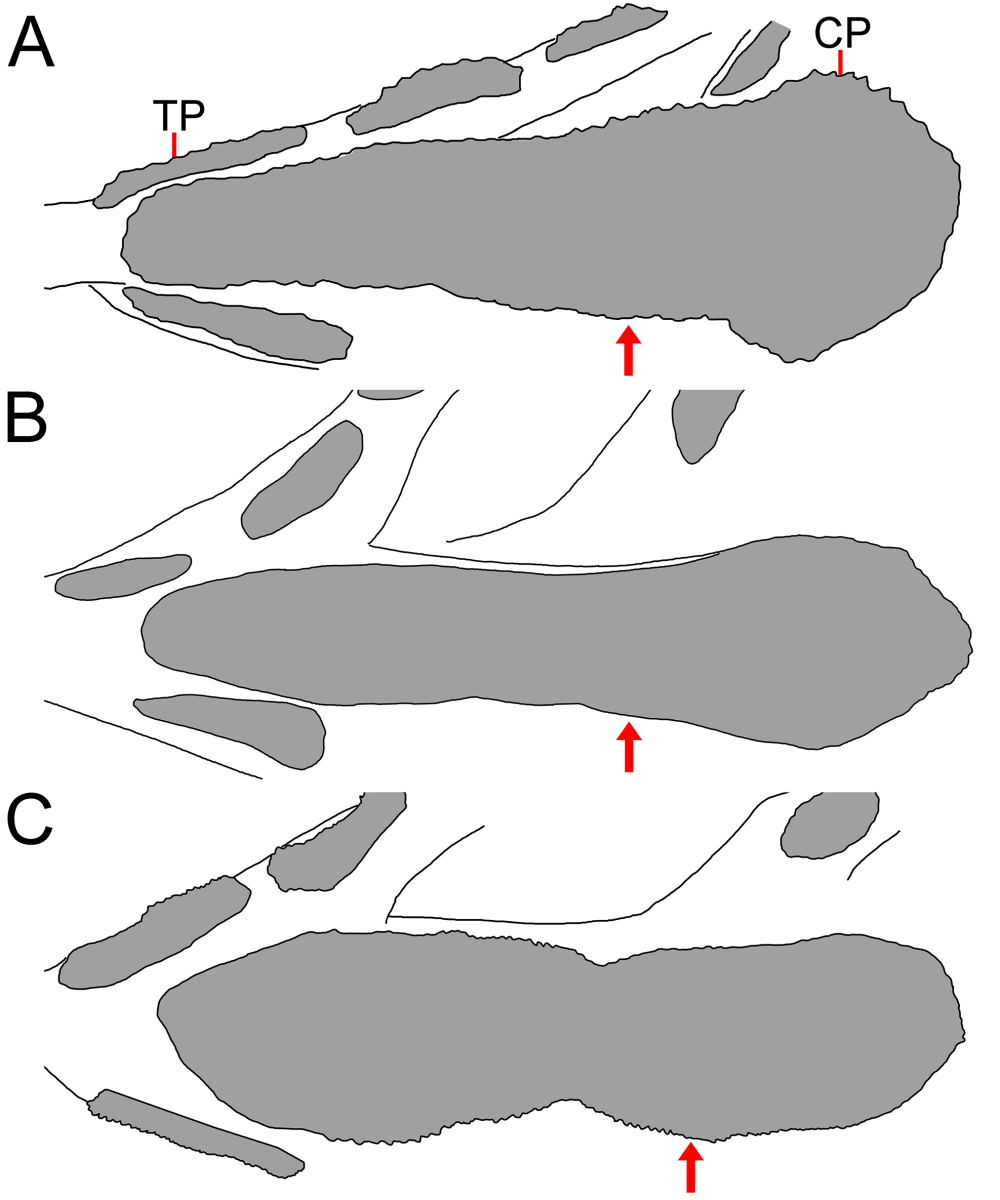
Copular tooth plates (CP) and anteriormost tooth plate (TP) on first gill arch of *Gyrinomimus johnsoni* sp. nov. **A**. Paratype, NSMT-P 49413, 315 mm SL; **B**. Holotype, USNM 283095, 194.0 mm SL; **C**. Paratype, SIO 70-306, 95.0 mm SL. Arrow indicates level of eye. Anterior to left. Figures not to scale.

Figs 12, 13, 14, 15, Tables 4, 5

Gyrinomimus sp. H.: Paxton 1989: 190 (mentioned).

##### New English name.

Johnson’s flabby whalefish.

**Table 4.** Meristic characters of *Gyrinomimus johnsoni* sp. nov. and *G. grahami* Richardson & Garrick, 1964. Paired characters are presented as left/right whenever available. Abbreviations: HT, holotype; NT, non-types; PT, paratypes.

	*G. johnsoni* sp. nov.		*G. grahami*
	This study		Paxton and Gon 1990
	HT	PT (*n* = 7)	HT; NT (*n* = 24)
Dorsal-fin rays	18	16–19	14–16
Pectoral-fin rays	17/17	17–21/18–20	21–22
Anal-fin rays	17	17–20	14–16
Principal caudal-fin rays	8 + 8	8 + 8	–
Vertebrae	33 + 22 = 55	30–32 + 22–24 = 53–55 (*n* = 5)	51–57 (usually 54–55)
Lateral-line scales	18/18	15–19/17	12–17 (usually 13–16)
Lateral-line pores	19/19	16–18/18	13–18 (usually 14–17)
GRI	3 + 1 + 5	1–2 + 1 + 2–4	1–2 + 1 + 2–4
GRII	1 + 1 + 2	1 + 1 + 2–4	–
GRIII	1 + 0 + 2	0 + 1 + 1–3	–
Medial GRI	4	2–3	2–5
Anal lappets	0	0	0
Head pores			
Mandibular	7	6–8	5–7
Preopercle	2	0–1+ (slightly damaged)	3–4
Infraorbital	7 + 0	7 + 0	7–8 (usually 8)
Supraorbital	6	6	6–7 (usually 7)
Main canal	5	4–6	4–5 (usually 4)
Supratemporal	1	1	1

**Table 5.** Morphometric characters of *Gyrinomimus johnsoni* sp. nov. and *G. grahami* Richardson & Garrick, 1964. Abbreviations: A, anal-fin; D, dorsal-fin; HT, holotype; NT, non-types; P, pectoral-fin; PT, paratypes; SD, standard deviation.

	*G. johnsoni* sp. nov.			*G. grahami*
	This study			Paxton & Gon, (1990)
	HT	PT (*n* = 7)		HT; NT (*n* = 24)
		Mean (range)	SD	Range
SL (mm)	194.0	95.0–315		66.4–310
% SL				–
HL	30.3	29.4 (27.1–32.0)	1.7	25.8–33.7
Head width	9.5	8.4 (6.7–9.7)	1.0	–
Eye diameter	0.8	1.1 (0.7–1.5)	0.3	–
Snout length	9.8	9.0 (8.0–10.5)	1.1	7.9–12.3
Upper-jaw length	25.0	24.2 (21.9–28.2)	2.2	21.9–30.7
Lower-jaw length	23.4	23.3 (20.3–26.6)	2.1	–
Posterior premaxilla to opercular margin	5.2	5.8 (4.6–8.6)	1.5	3.5–7.0
Body depth	18.4	20.6 (17.4–25.4)	3.3	–
Anus to anal-fin origin	1.9	2.2 (1.1–2.9)	0.7	–
D–A	15.9	17.9 (15.7–22.4)	2.7	9.3–14.8
Predorsal length	71.3	67.9 (65.4–69.7)	1.7	–
Prepectoral length	27.9	29.7 (27.1–34.5)	2.4	–
Preanal length	69.5	66.4 (62.9–70.7)	2.9	71.6–78.6
D base	17.1	20.1 (17.8–22.4)	1.6	–
A base	17.1	20.9 (19.6–22.7)	1.3	11.0–13.7
P length	7.5	8.3 (5.5–10.1)	1.6	–
Caudal-peduncle depth	5.3	6.0 (5.1–7.6)	0.9	–
Caudal-peduncle length	15.0	14.2 (11.5–16.5)	1.9	9.0–14.2
Copular tooth plate length	8.8	8.7 (7.6–9.5)	0.7	7.2–10.3
Copular tooth plate width	2.1	2.4 (1.9–2.9)	0.4	–
Gill filament on first arch	3.0	3.2 (2.4–4.1)	0.7	–
Gill filament on fourth arch	0.5	~0.2	N/A	–
% Gill filament on first arch				–
Gill filament on fourth arch	17.2	~6.3	N/A	–
Length/width ratio of copular tooth plate	4.2	3.8 (3.2–4.7)	0.6	3.0–4.4

##### New Standard Japanese name.

Hoppo-kujirauo (ほっぽうクジラウオ).

##### Type material.

***Holotype***. • USNM 283095, 194.0 mm SL, off California, U.S.A., northeastern Pacific, 39°46'18.1"N, 128°00'28.8"W, 0–2315m, *Golden Fleece*, 11 Dec. 1981, coll. D. L. Stein. ***Paratypes***. Seven specimens, 95.0–315 mm SL. • AMS I.26871-001 (formerly OSUO 1520), 124.5 mm SL, northeastern Pacific, 52°52'12"N, 163°03'00"W, 0–2350 m, 10 ft MPS2, 0707–1330 hrs, 6 Aug. 1966, coll. D. L. Stein. • BCPM 79-11242, 132.9 mm SL, northwestern Pacific, 50°N, 145°E, no other data. • NSMT-P 49413 (formerly TH 841463), 315 mm SL, 32°13'N, 158°11'E, northwestern Pacific, 0–775 m, R/V *Kaiyo-maru*, operation number KMT-3, station E., KMT net, 0932–1121 hrs, 29 May 1984. • NSMT-P 71817 (formerly TH 840886), 109.1 mm SL, 31°41'00"N, 157°39'00"E, northwestern Pacific, 0–2320 m, R/V *Kaiyo-maru*, operation number KMT-10, station E., KMT net, 1227–1701 hrs, 1 Jun. 1984. • SIO 70-306, 95.0 mm SL, off Japan, northwestern Pacific, 32°09'33"N, 136°05'4.5"E–31°50'23.99"N, 136°19'48"E, 0–1400 m, R/V *Melville*, cruise Antipode IV, station 51A-Tr #1, 10-ft IKMT net, 28–29 Aug. 1970, coll. R. H. Rosenblatt & SIO party. • USNM 296934, 290.0 mm SL, off Humboldt County, just north of Eel River Canyon, California, U.S.A., northeastern Pacific, 40°41'30.1"N, 124°32'31.2"W, 366 m, *Stephanie*, 30 May 1988, coll. P. Enberg. • ZMH-ICH-0028917 (formerly ISH 53-1986 and OSUO 2374), 97.7 mm SL, off Oregon, U.S.A., northeastern Pacific, 44°52'30"N, 125°54'42"W, 1937–2320 m, *YAQUINA*, cruise y7505D, station NH-65, Haul 2329, 8’ MT net, 25 May 1975.

##### Non-type.

AMS I.29640-001 (formerly TH 840845), 147.2 mm SL, 12°34'12"N, 151°51'00"E, western Pacific, 0–1410 m, R/V *Kaiyo-maru*, 0710–1045 hrs, 14 Feb. 1986.

##### Diagnosis.

A species belonging to *G. grahami* species group and differs from all congeners in having: medial tooth patch on first gill arch 2–4; POP 0–2; IO 7 + 0 = 7; inner ~ 3–5 rows of teeth in the anterior jaws in diagonal rows (in specimens < ~ 150 mm SL); anterior margin of anteriormost GRI reaching near anterior margin of the copular tooth plate, often before anterior margin of copular tooth plate; cavernous tissue only surrounding anus; anal lappets absent; filaments on fourth gill arch tiny or nearly absent, length < 17.3% filament on first arch; cavernous tissue, when discernable, only around anus; vertebrae 30–33 + 22–24 = 53–55; dorsal-fin rays 16–19; anal-fin rays 17–20; lateral-line pores 16–19; lateral-line scales 15–18; and copular tooth plate rather slender, greatest width 3.2–4.7 in length.

##### Description.

Meristic and morphometric characters are provided in Tables 4, 5. Data of holotype are provided first, followed by those of paratypes in parentheses whenever available. Dorsal-fin rays 18 (16–19); pectoral-fin rays 17/17 (17–21/18–20); anal-fin rays 17 (17–20); principal caudal-fin rays 8 + 8 (7–8 + 7–8). GRI 3 + 1 + 5 (1–2 + 1 + 2–4); GRII 1 + 1 + 2 (1 + 1 + 2–4); GRIII 1 + 0 + 2 (0–1 + 1 + 1–3); medial GRI 4 (2–3). Lateral-line pores 19/19 (16–18/18); lateral-line scales 18/18 (15–17/17). Head pores (Fig. 13): MD 7 (6–8); POP 2 (0–1+; slightly damaged); IO 7 + 0 = 7 (7 + 0 = 7); SO 6 (6); M 5 (4–6); ST 1. Vertebrae 33 + 22 = 55 (30–32 + 22–23 = 53–54; *n* = 5).

Body rather slender and compressed, depth at pectoral-fin base 5.4 (3.9–5.8) in SL. Head moderate and slightly compressed, length 3.3 (3.1–3.7) in SL. Dorsum and abdomen with distinct central ridge. Nostrils similar to head lateral-line pores, closer to tip of snout than eye; anterior nostril slightly smaller than posterior one; both nostrils oval; nasal organ mostly in anterior nostril. Eye tiny, diameter 36.3 (21.2–44.5) in HL. Interorbital broadly concave, without distinct pits; anterolateral projection of frontal slightly posterior to eye. Caudal peduncle rather short and deep, length and depth 5.8 (4.4–5.9) and 2.0 (1.7–2.6) in HL, respectively. Cavernous tissue surrounding anus (Fig. 14); present on mouth roof, esophagus, and body wall behind fourth gill arch. Anal lappets absent.

Mouth enormous and oblique; upper-jaw length 1.2 (1.1–1.4) in HL; lower jaw with two posterior spines, with ventral spine blunt and lateral spine blunt or indistinct and covered under skin. Jaw teeth elongated, with inner ~ 3–5 rows in diagonal rows in specimens <150 mm SL (all 5 rows of anterior teeth in diagonal rows in SIO 70-306 and ZMH-ICH-0028917), others in distinct longitudinal rows; inner row of teeth largest; premaxillary and dentary symphyses edentate. Upper jaw with 10 (4–11) rows of teeth anteriorly, its number slightly decreases posteriorly, with 6 (1–5) rows posteriorly. Lower-jaw teeth similar to those of upper jaw, with 10 (4–13) rows anteriorly and 5 (2–5) rows posteriorly. Vomerine, palatine, and ectopterygoid teeth similar in shape to jaw teeth. Vomerine tooth patch domed, with 5 (3–8) rows of teeth. Palatine tooth patch beginning at level of middle of vomer and separated by large gap, with 6 (2–9) rows of teeth; its anterior end anterior to level of eye. Ectopterygoid tooth patch broad, with ~ 5 (2–10) teeth rows and nearly attached to palatine tooth patch; its origin posterior to palatine, extending posteriorly beyond rictus and before end of premaxilla. Copular tooth patch (Fig. 15) teardrop shaped, its anterior margin pointed, with rather straight or slightly concave lateral side, and slightly widens posteriorly; its length and width 3.4 (3.0–3.8) and 14.3 (9.9–15.8) in HL, respectively; length/width ratio 4.2 (3.2–4.7).

Three free gill arches, with small slit behind ventral arm of third arch near angle, extending ~ 1/2 (1/2 to entire) length of filament-bearing part of ventral arm; dorsal arm of arch I 1/2 (1/2) free; dorsal arm of arch II 1/4 (1/2) free. Gill filaments of anterior three arches moderately long and well developed; those on arch IV tiny to short, length of filaments on arch IV 17.2 (too tiny to measure, ≤ 6.3)% length of filaments on arch I. Gill rakers forming plates on lateral face of outer three arches and medial face of arch I; present on outer face of arch IV and medial face of arch II in partial specimens. Anterior margin of anteriormost GRI reaches near anterior margin of copular tooth plate, before anterior margin of copular tooth plate in most specimens. Two dorsal pharyngeal tooth plates, both domed and slightly oval; no ventral pharyngeal tooth plate.

Lateral-line pores large, most pores almost as wide as canal (≤ 3/4 width of canal), with last pore ~ 3/4 width of canal. Lateral line usually without flaps anterior to pores on posterior portion (present in one specimen); keels absent (present in one specimen). Two papillae on both anterior and posterior margins of pores. No papillae on base of caudal fin. Dorsal accessory line of neuromasts beginning slightly before level of upper limit of gill opening, with ~ 15 (*n* = 1) papillae scattered on dorsum, and extending posteriorly 2/3 of way to dorsal-fin origin, before mid-dorsal keel. Lateral-line scales without spines on dorsal and ventral arms and not penetrate skin of lateral-line canal.

Lateral-line system of head well developed. Head with numerous scattered papillate superficial neuromasts, ~ 0–12 (broken in holotype) papillae on tip of snout between midline and first supraorbital pore; 4 (1–3) papillae above anterior nostril; 4 (4–6) papillae posteroventral to anterior nostril; 2–4 (broken in holotype) papillae dorsal to first infraorbital pore; 0 (0) papillae dorsal to second and third infraorbital pores; ~ 11 (10 ) papillae dorsal to first and second main-canal pores; 2 (1) papillae medial to supratemporal pore; 2 (6–11) papillae anterior to preopercular pores (or preopercle and cheek when pores absent); ~ 4 (6) papillae on opercle; 11 (9–23) papillae scattered above mandibular pores, becoming denser posteriorly.

Dorsal and anal fins far back on body, with dorsal-fin origin slightly posterior to that of anal fin; their tips pointed posteriorly. Dorsal fin with ~ 8^th^–16^th^ (7^th^–17^th^; *n* = 3) rays branched, others unbranched. Anal fin with middle ~ 6^th^–15^th^ (7^th^–18^th^; *n* = 2) rays branched, others unbranched. Dorsal- and anal-fin rays branched at tip, and becoming proximally as growth. Pectoral fin low, broad-based, directed posterodorsally, and rather short, length 4.0 (2.8–5.6) in HL; middle rays longest; all rays simple. Subpectoral organ only discernable in SIO 70-306.

##### Coloration.

When preserved (Fig. 12), body dark to light brown. Gill arches, rakers and filaments, and inner face of operculum pale. Some black patches occasionally present on gill arches and hyoid arches. Teeth transparent. Peritoneum and stomach black. Esophagus and mouth roof with dark marble patterns. Abdomen with dark reticulated pattern.

##### Distribution.

Currently known from specimens collected in the northern Pacific Ocean between 12°N and 53°N.

##### Etymology.

We are pleased to name this species after the late Dr. G. David Johnson, National Museum of Natural History (USNM), for his great contributions to our knowledge of cetomimids, and for his encouragement in our study.

The standard Japanese name Hoppo-kujirauo (ほっぽうクジラウオ) is here proposed based on SIO 70-306. “Hoppo” means northern, indicating the northern distribution of the species, in contrast to its most similar congener, *G. grahami*, which is cosmopolitan in the Southern Hemisphere.

##### Remarks.

The non-type specimen (AMS I.29640-001) has 59 total vertebrae, which is distinctly higher than other specimens examined in this study (vs 53–55; *n* = 6). However, other characters, such as the distribution of cavernous tissue, position of the anteriormost GRI, and the inner rows of teeth on both jaws arranged in diagonal rows, are consistent with the type specimens. Additional specimens are required to determine whether this discrepancy falls within the natural range of variation for this species, given that vertebral counts in *Cetostoma regani* Zugmayer, 1914 are known to range from 47 to 53 (Paxton 1989).

### Key to the species group, valid species, and new species of *Gyrinomimus*

**Table:** 

1	Gill filaments of fourth gill arch moderate to large, > 40% length of first-arch filaments; anal lappets present over anal-fin base (unknown in *G. andriashevi*) (*G. bruuni* species group)	**2**
–	Gill filaments of fourth gill arch tiny to small, < 34.5 % length of first-arch filaments; anal lappets absent	**6**
2	Dorsal- and anal-fin rays 14–15; lateral-line pores 17	**3**
–	Dorsal- and anal-fin rays 17–21; lateral-line pores 19–25	**4**
3	Medial GRI 4; cavernous tissue from anus to base of third anal-fin ray; total vertebrae 54; copular tooth plate rather broad, greatest width 2.1 in length	***Gyrinomimus alepis* sp. nov**.
–	Medial GRI 0; cavernous tissue over anus and a separated small patch over first anal-fin ray; total vertebrae 58; copular tooth plate elongated, greatest width 3.4 in length	***Gyrinomimus andriashevi***
4	Lateral-line scales with 2–5 (very rarely 1) dorsal and ventral spines and protruding from skin (Fig. 6B); anal lappets 8–14; IO 7 + 2 (8 + 2 in one side of one specimen); GRIII 1 + 1 + 1; total vertebrae 51–53; copular tooth plate relatively long and narrow, greatest width 3.3–4.1 in length (Fig. 7)	***Gyrinomimus amaokai* sp. nov**.
–	Lateral-line scales with 0–1 dorsal and ventral spines and not protruding from skin; anal lappets 2–9; IO 8 + 2 (7 + 2 in one specimen of *G. bruuni*); GRIII 0 + 1 + 1 (1 + 1 + 1 in two specimens of *G. bruuni*); total vertebrae 54–58; copular tooth plate relatively short and broad, greatest width 2.0–3.1 in length	**5**
5	Cavernous tissue at anus and extending to over base of anal-fin ray 6–21; anal lappets 4–9, with the last over base of anal-fin ray 7–15; copular tooth plate moderately long and broad, greatest width 2.5–3.1 in length (Fig. 11)	***Gyrinomimus johnpaxtoni* sp. nov**.
–	Cavernous tissue at anus and extending to over base of anal-fin ray 1–3; anal lappets 2–6, with the last over base of anal-fin ray 4–6; copular tooth plate very short and broad, greatest width 2.0–2.4 in length (Fig. 16C)	***Gyrinomimus bruuni***
6	Tooth plates on medial face of ventral arm of first gill arch present; cavernous tissue, around anus and sometimes with an additional separate patch posterodorsal to anus (Figs 14, 17B; *G. grahami* species group)	**7**
–	No tooth plates on medial face of first gill arch; cavernous tissue around anus only or with additional patches elsewhere, but never only a second patch posterodorsal to anus (*G. myersi* species group)	**8**
7	All jaw teeth in longitudinal rows; anterior margin of anteriormost GRI far posterior to anterior margin of copular tooth plate (Fig. 17C); dorsal and anal-fin rays 14–16; cavernous tissue around anus and a separate patch posterodorsal to anus; POP 3–4	***Gyrinomimus grahami***
–	Inner 3–5 tooth rows on anterior jaws in diagonal rows in specimens < ~ 150 mm SL; anterior margin of anteriormost GRI reaching near anterior margin of copular tooth plate, often extending before (Fig. 15); dorsal- and anal-fin rays 17–19 (16 in one specimen); cavernous tissue, only around anus; POP 0–2	***Gyrinomimus johnsoni* sp. nov**.
8	No cavernous tissue at dorsal-fin origin or isthmus; keels between posterior lateral-line pores small to moderate in specimens over 150 mm SL	***Gyrinomimus parri***
–	Cavernous tissue at dorsal-fin origin and usually at isthmus; keels between posterior lateral-line pores large in specimens over 100 mm SL	***Gyrinomimus myersi***

## Discussion

### Comparison of *G. alepis* with congeners

The presence of long gill filaments on the fourth gill arch and anal lappets indicate *G. alepis* is a member of the *G. bruuni* species group (vs filaments on fourth gill arch tiny to moderate and anal lappets absent). It differs from all other species in the group except *G. andriashevi* by having anal lappets lacking scales and rudimentary (vs anal lappets with scales and well developed), medial GRI 4 (vs 0; Table 1), dorsal-fin rays 15 (vs 17–21). In addition, *G. alepis* is most similar to *G. andriashevi*, both sharing same counts of dorsal and anal-fin rays. However, *G. alepis* differs from *G. andriashevi* in having: medial GRI 4 (0 in *G. andriashevi*; Table 1); GRI 2 + 1 + 8 (vs 1 + 1 + 2–3); total vertebrae 54 (vs 58); cavernous tissue from anus to base of third anal-fin ray (vs over anus and a separated small patch over first anal-fin ray); and length/width ratio of copular tooth plate 2.1 (vs 3.4; Table 2).

**Figure 16.**
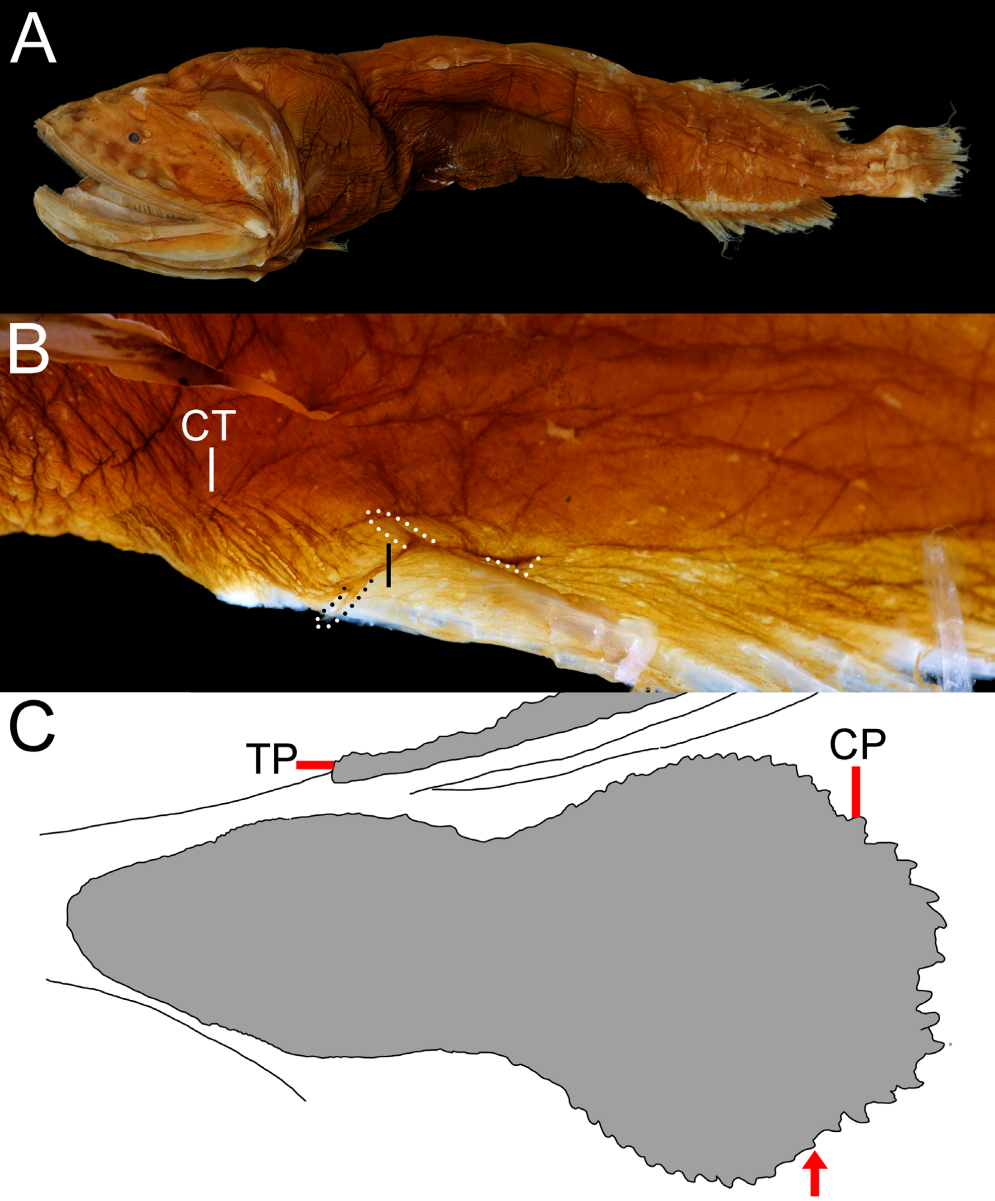
Preserved specimen of *Gyrinomimus bruuni* Rofen, 1959. **A** Lateral view, SIO 69-350, 116.9 mm SL; **B**. Close-up image (left-right reversed) of cavernous tissue (CT) around anus, with its posterior extension indicated by black bar, and anal lappets (circled). Same specimen as **A**; **C**. Illustration of copular tooth plate (CP) and anteriormost tooth plate (TP) on first gill arch, ASIZP 65438, 140.6 mm SL. Arrow indicates level of eye. Photo by Y.-C. Hsu (**A**) and Y.-C. Fan (**B**). Anterior to left. Figures not to scale.

**Figure 17.**
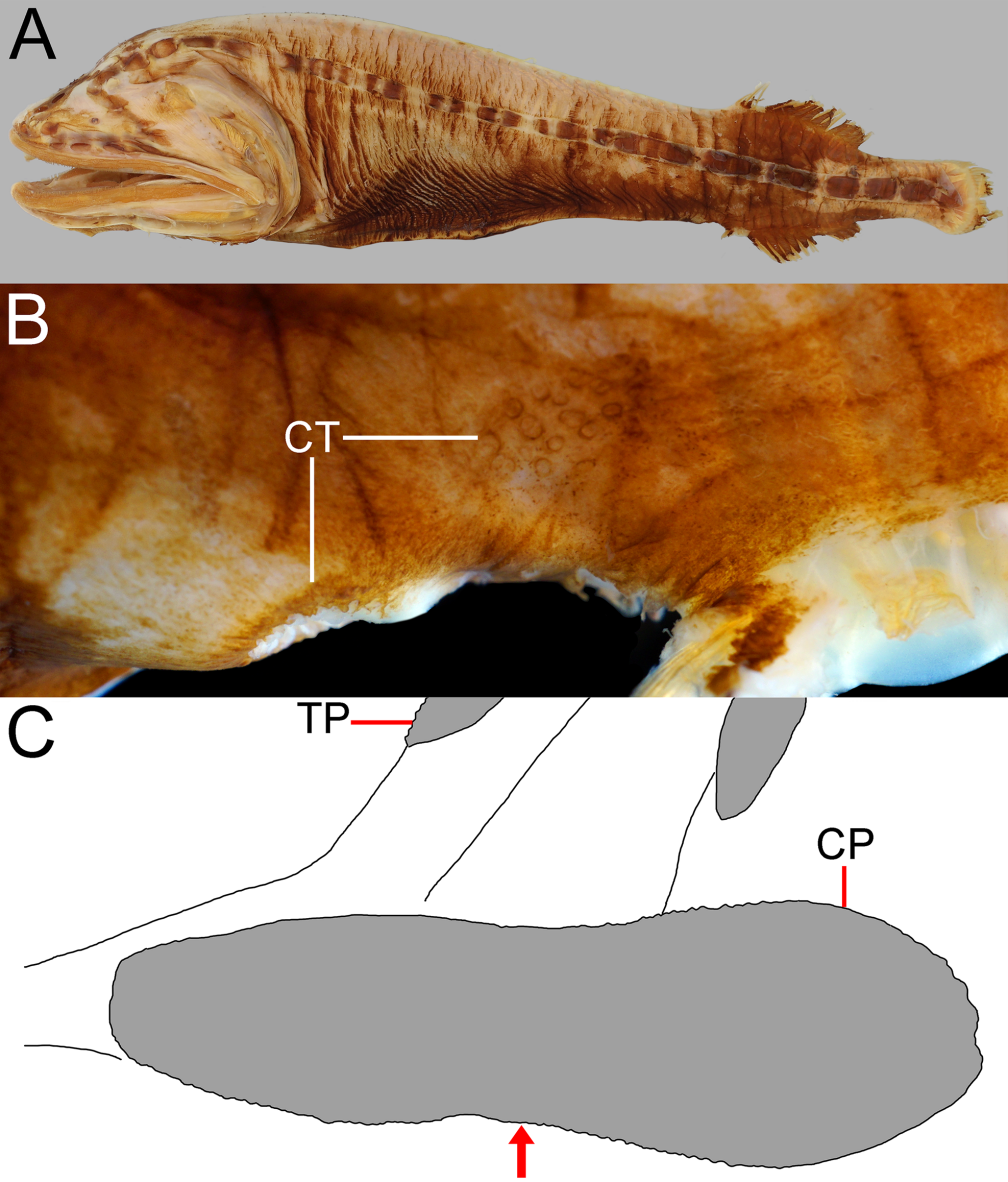
Preserved specimen of *Gyrinomimus grahami* Richardson & Garrick, 1964, LACM 10684-1, 207.0 mm SL. **A**. Lateral view; **B**. Close-up image of cavernous tissue around anus; **C**. Illustration of copular tooth plate (CP) and anteriormost tooth plate (TP) on first gill arch. Arrow indicates level of eye. Photo by Y.-C. Hsu (**A**) and Y.-C. Fan (**B**). Anterior to left. Figures not to scale.

### Comparison of *G. amaokai* with congeners

The presence of moderate to long filaments on the fourth gill arch and anal lappets indicates *G. amaokai* is a member of the *G. bruuni* species group. *Gyrinomimus amaokai* is unique within the *G. bruuni* species group, as well as congeners in having 2–5 (very rarely 1) dorsal and ventral spines on lateral-line scales that penetrates skin of lateral-line canal (Fig. 6B). Moreover, such characteristic is currently only seen in this species within the entire Cetomimidae (Paxton 1989; Su, unpubl. data).

Despite the spines on the lateral-line scales, and among the *G. bruuni* species group, *G. amaokai* is most similar to *G. johnpaxtoni* and *G. bruuni* in having anal lappets well-developed and with scales (vs rudimentary and without scales in *G. alepis*), dorsal-fin rays 17–21 (vs 14–15 in *G. alepis* and *G. andriashevi*; Table 1), dorsal-fin base 15.2–19.9% SL (vs 13.1–13.9% SL; Tables 2, 3), anal-fin base 14.5–17.8% SL (vs 12.7–13.9% SL), and length/width ratio of copular tooth plate 3.3–4.1 (vs 2.1 in *G. alepis*). Moreover, *G. amaokai* differs from *G. johnpaxtoni* and *G. bruuni* in having total vertebrae 51–53 (vs 54–58; Table 1); GRIII 1 + 1 + 1 (vs usually 0 + 1 + 1, except for 1 + 1 + 1 in two specimens of *G. bruuni*); IO 7 + 2 (8 + 2 in one side of one specimen) (vs usually 8 + 2, 7 + 2 in one specimen of *G. bruuni*); anal lappets 8–14 (vs 2–9); length/width ratio of copular tooth plate 3.3–4.1 (vs 2.0–3.1 in *G. johnpaxtoni* and *G. bruuni*; Table 3).

### Comparison of *G. johnpaxtoni* with congeners

The presence of long gill filaments on the fourth gill arch and anal lappets indicates *G. johnpaxtoni* is a member of the *G. bruuni* species group. Among species in this group, *G. johnpaxtoni* differs from *G. andriashevi* and *G. alepis* in having dorsal-fin rays 19–21 (vs 14–15 in both species; Table 1) anal-fin rays 18–21 (vs 14–15), lateral-line pores 19–25 (vs 17), and medial GRI 0 (vs 4 in *G. alepis*). *Gyrinomimus johnpaxtoni* differs from *G. amaokai* in having total vertebrae 54–58 (vs 51–53 in *G. amaokai*; Table 1), GRIII 0 + 1 + 1 (vs 1 + 1 + 1), IO 8 + 2 (vs 7 + 2, very rarely 8 + 2), length/width ratio of copular tooth plate 2.5–3.1 (vs 3.3–4.1; Table 3), and spines on dorsal and ventral margins of lateral line 0–1, not piercing from skin of canal (vs spines 1–5, and distinctly piercing out of the skin of canal).

*Gyrinomimus johnpaxtoni* is most similar to *G. bruuni* (Fig. 16A), sharing similar meristic and morphometric characters (Tables 1, 3), and once been confused (Misawa et al. 2024). However, *G. johnpaxtoni* differs from *G. bruuni* in having cavernous tissue at anus extending to over base of anal-fin ray 6–21 (vs 1–3; Figs 10, 16B), the last anal lappet over base of anal-fin ray 7–15 (vs the last over base of anal-fin ray 4–6), and copular tooth plate rather elongated, length/width ratio 2.5–3.1 (vs rather short and broad, length/width ratio 2.0–2.4; Table 3; Figs 11, 16C).

Despite the morphological differences, the COI sequences of paratype (HUMZ 234625; LC799418) and the sequence of *G. bruuni* (ASIZP 69735; FTWS392-09) show 5.5% distance calculated by K2P model, thereby confirming the discrimination of the two species.

### Comparison of *G. johnsoni* with congeners

The absence of anal lappets, presence of rakers on medial face of first gill arch, and gill filaments on fourth gill arch tiny indicates that *G. johnsoni* is a member of the *G. grahami* species group (vs anal lappets present and filament on fourth gill arch well developed in *G. bruuni* species group, no medial rakers on first arch in *G. myersi* species group and most members of *G. bruuni* species group). It differs from *G. grahami* (Fig. 17A), the only other nominal species within this group, in having dorsal-fin rays 17–19, rarely 16 (vs 14–16 in *G. grahami*; Table 4; Paxton 2015), POP 0–2 (vs 3–4), cavernous tissue, forming single patch (vs around anus and an additional separated patch posterodorsal to anus; Figs 14, 17B), anterior margin of anteriormost GRI reaching anterior 1/3 of copular tooth plate, beyond anterior margin of copular tooth plate in most specimens (vs reaching approx. middle of copular tooth plate; Figs 15, 17C), and inner ~ 3–5 rows of teeth on anterior jaws in diagonal rows in specimens <~150 mm SL (vs all jaw teeth in longitudinal rows in all sizes).

### Comparison of larval or male cetomimids

As mentioned above, two families, Mirapinnidae and Megalomycteridae are now recognized as larval and male cetomimids. Among the ten species once included in these two families, *Parataeniophorus gulosus* Bertelsen & Marshall, 1956 and *Cetomimoides parri* Koefoed, 1955 are now junior synonyms of *Cetostoma regani*; *Parataeniophorus bertelseni* Shiganova, 1989 is a junior synonym of *Ditropichthys storeri* (Goode & Bean, 1895); *Mirapinna esau* Bertelsen & Marshall, 1956 is a senior synonym of *Procetichthys kreffti* Paxton, 1989 (Johnson et al. 2009; Paxton et al. 2016). Based on their original descriptions and JP’s unpublished data, we here compare the remaining species with our new species.

The holotype of *Parataeniophorus brevis* Bertelsen & Marshall, 1956 possesses 15 dorsal- and anal-fin rays, ~16 pectoral-fin rays, and 45 total vertebrae. *Megalomycter teevani* Myers & Freihofer, 1966 is also similar to *P. brevis* in having 45 total vertebrae but having 16 dorsal-fin and 17 anal-fin rays. Such combination is only seen in female specimens of *Danacetichthys galathenus* Paxton, 1989 and *Ramphocetichthys savagei* Paxton, 1989.

The holotype of *Eutaeniophorus festivus* (Bertelsen & Marshall, 1956) possesses 20 dorsal-fin rays, 18 anal-fin rays, 23 pectoral-fin rays, 10 + 9 principal caudal-fin rays, 2 + 8 gill rakers, and 56 total vertebrae. Such fin-ray and vertebral counts indicate that it is possibly a larva of *Cetomimus* or *Gyrinomimus* (Paxton 1989). The numbers of dorsal- and anal-fin rays and vertebrae overlapped with *G. johnpaxtoni* and *G. bruuni* in this study. However, *E. festivus* differs from the two aforementioned species in having more pectoral-fin rays (23, vs 16–21) and number of principal caudal-fin rays (10 + 9, vs 7–8+7–8).

The holotype of *Ataxolepis apus* Myers & Freihofer, 1966 possesses 16 dorsal-fin rays, 16 anal-fin rays, 20 pectoral-fin rays, and 49 total vertebrae. Similarly, the holotype of *Ataxolepis henactis* Goodyear, 1970 possess 15 dorsal-fin rays, 17 anal-fin rays, 21 pectoral-fin rays, and 48 total vertebrae. The combination of fin-ray counts and vertebrae number also indicate that they are possibly male specimens of *Cetomimus* or *Gyrinomimus* (Paxton 1989). Both species differ from the new species described here in having 48–49 vertebrae (vs > 50 in all species).

Lastly, the holotype of *Vitiaziella cubiceps* Rass, 1955 possesses 16 dorsal-fin rays, 17 anal-fin rays, 19 pectoral-fin rays, and 54 total vertebrae. Such combination of fin-ray counts and vertebrae number also indicate that it is a male specimen of *Cetomimus* or *Gyrinomimus* (Paxton 1989). The combination is either similar or overlapped with *G. alepis*, *G. bruuni*, *G. grahami*, *G. johnsoni*, and *G. myersi* (Paxton and Gon 1990; this study), as well as some species of *Cetomimus* (Paxton, unpubl. data). As the diagnostic characters, such as the number of lateral-line pores/scales, cavernous tissue are absent in male specimens, further study is needed to confirm the identity of *V. cubiceps*.

Although molecular approaches are expected to play an important role in clarifying the identities of larval and male specimens, their effectiveness ultimately depends on the correct identification of reference material and the recognition of robust diagnostic characters. In several cases, such characters remain poorly defined. For example, the type series of *E. festivus* likely includes more than one species, as indicated by the wide range of reported vertebral counts (47–55; Bertelsen and Marshall 1956), suggesting that larval specimens of different species are morphologically very similar. We acknowledge that the new species described herein could potentially be conspecific with taxa previously established solely on the basis of larval or male materials, particularly *V. cubiceps* (see above). Nevertheless, we emphasize that the description of species based on adult females is both justified and necessary. Adult females provide the most stable and informative set of diagnostic characters and are therefore indispensable for reliably linking larval, male, and female stages within Cetomimidae, as well as for establishing a coherent and taxonomically stable framework for the family.

### Comments on morphological characteristics

Paxton (1989) described several characters in distinguishing species of *Gyrinomimus*, including the numbers of anal lappets, lateral-line pores and scales, spines on lateral-line scales, gill rakers, dorsal- and anal-fin rays, and vertebrae, distribution of cavernous tissue, and the size of filaments of fourth gill arch.

Based on our examination, in addition to the aforementioned characters, the extent and distribution of anal lappets, the number of pores in the head canals of the lateral-line system, the extent of flaps and keels on the posterior lateral-line canal, the length/width ratio of the copular tooth plate are useful to distinguish between species. On the other hand, the numbers of pectoral-fin rays, papillae on head and body, and most morphometric characters are either variable or highly overlapped and therefore are of little value to distinguish species.

### Distribution pattern

As mentioned by Paxton (1989), most cetomimid specimens were collected by open net, and therefore the precise collection depth is unknown. While the majority of specimens examined here span a wide range of depths, several notable bathymetric anomalies provide insights into their ecology.

The holotype of *G. amaokai* was collected via gill net at depth 350–460 m, considerably shallower than other specimens (known depth ~1000 m). Similariy, the holotype of *G. notius* (= *G. grahami*) was also collected from exceptional shallow waters (445–470 m). Fedorov and Balushkin (1983) pointed out that the hydrological conditions in the Ross Sea allow deep-water fishes to penetrate into shallower slope waters. We suggest that this phenomenon may be true for the waters off northeastern Hokkaido, where canyons of the Okhotsk Sea have the 2000 m contour within 20 km of shore and 1000 m within 15 km of shore. However, local fishermen in the area have not reported any other unusual deep-sea fishes in their nets (K. Amaoka, pers. comm., Jul 1996).

The evidence of vertical migration is also apparent in some specimens examined in this study, which was also found in *Cetostoma regani* and *Ditropichthys storeri* by Paxton (1989). The paratype of *G. johnpaxtoni*, two specimens of *G. bruuni*, and one specimen of *G. myersi* housed in ASIZP were collected by local fishermen as bycatch of Sakura shrimp (*Lucensosergia lucens*) fishery in the northeastern Taiwan at depths less than 500 m. Based on our observation, the vessels depart at midnight for fishing and return at noon, suggesting these fishes ascend into shallower waters nocturnally in search of prey.

Most species of *Gyrinomimus* show circumglobal distribution (Paxton 1989, 2002; Paxton et al. 2016; this study). However, all of the specimens of *G. johnsoni* were collected from the northern Pacific, in contrast with its close relative, *G. grahami*, which is cosmopolitan in the South Hemisphere (Paxton and Gon 1990; Paxton 2015). Additionally, *G. amaokai* appears to be cosmopolitan in the North Hemisphere.

## Comparative materials

*Eutaeniophorus festivus*: Holotype: • ZMUC P.2329853, 49.9 mm SL, eastern Indian Ocean, 4°02'S, 95°44.5'E, ca 70 m, *Dana*, station 3853 III, 16 Oct. 1929.

*Gyrinomimus andriashevi*. Holotype: • ZIN 47725, 231.8 mm SL, Lazarev Sea, Antarctic Ocean, 65°20'S, 2°34'E, 1310–1360 m over bottom depth of 1370–1400 m, *Vol’nyy Veter*, station 14, 20 Jan. 1983, coll. I. A. Trunov.

*Gyrinomimus bruuni*. Holotype: • ZMUC P.23452, 83.5 mm SL, off Kenya, 05°25'S, 47°09'E, 4820 m, *Galathea*, expedition 1950–1952, station 234, 10 Mar. 1951. Non-types: AMS I.29639-001 (formerly TH 861084), 192.1 mm SL, 12°35'N, 151°53'E, 0–1711 m, R/V *Kaiyo-maru*, 0620–1021 hrs, 15 Feb. 1986. • ASIZP 65438, 140.6 mm SL, off Daxi fishing port, Taiwan, 300–500 m, bottom trawl, 29 Jan. 2005, coll. H.-C. Ho. • ASIZP 69735, 148.6 mm SL, off Daxi fishing port, Taiwan, bottom trawl, 5 Feb. 2007, coll. P.-F. Lee and J-.Y. Tsai; BOLD system accession number: FTWS392-09. • NSMT-P 68765, 85.4 mm SL, south China Sea, 14°39'N, 114°07'E, 0–1380 m, R/V *Hakuho-maru*, operation number KH81-5, station 6, 0044–0337 hrs, 10 Feb. 1978. • SIO 69-350, 116.9 mm SL, eastern Pacific, 1°25'N, 86°W, R/V *Thomas Washington*, cruise Piquero VIII, station 2A & 2B, 10-ft IKMT net, 2 Jul. 1969, coll. M. Barnett. • ZMUC P.2340009, 93.9 mm SL, 500 km east of French Guiana, western Central Atlantic, 7°22'N, 46°51'W, R/V *Dana*, expedition 1921, 14 Nov. 1921.

*Gyrinomimus grahami*: Holotype: • NMNZ P.003744, 66.8 mm SL, South of Cape Palliser, New Zealand, 42°07'S, 174°57'E, 1300 fathoms (= 2377 m), *V.U.Z*., station 58, bait trap, 31 Mar. 1956. Non-types: • LACM 10682-1, 207 mm SL, southeastern Pacific, 60°55'S, 114°47'W, R/V USNS *Eltanin*, 6 Jan. 1964. • LACM 11329-2, 171.7 mm SL, off New Zealand, Tasman Sea, 47°S, 162°47'W, R/V USNS *Eltanin*, 12 Dec. 1966.

*Gyrinomimus myersi*. Holotype: • YCM 2111, 99.2 mm SL, Tongue of the Ocean, Bahamas, 23°39'N, 76°41'W, 7000 feet wire out, Pawnee 1927, station 18, 18 Mar. 1927. Non-types: • ASIZP 62951, 88.9 mm SL, off Daxi fishing port, Taiwan, 11 Nov. 2002, coll. H.-C. Ho. • ASIZP 74774, 109.0 mm SL, off northeastern Taiwan, northwestern Pacific, 24°25'25.7"N, 122°12'23.8"E–24°20'48.1"N, 122°15'15.8"E, 1188–1209 m, R/V *Ocean Researcher I*, cruise 657, station CD199, otter trawl, 12 Sep. 2002, coll. K.-T. Shao. • HUMZ 120899, 210.9 mm SL, off west Greenland, north Atlantic, 63°36'17.99"N, 56°22'18"W–63°34'42"N, 56°23'18"W, 1344–1367 m, R/V *Shinkai-maru*, bottom trawl, 7 Aug. 1991. • MCZ 43332, 105.1 mm SL, western Indian Ocean, 5°55'30"S, 65°10'30"E, 0–275 m, R/V *Anton Bruun 06*, station 340A, 10-ft IKMT net, 1045–1830 hrs, 31 May 1964. • MCZ 165979, 110.5 mm SL, Bear Seamount, Georges Bank, northwest Atlantic, 39°52'40"N, 67°37'02"W, 0–2172 m, R/V *Delaware II 06-11*, IYGPT, 1045–1830 hrs, 14 June 2006. • NSMT-P 49415 (formerly TH 820233), 333 mm SL, northwestern Pacific, 30°14'N, 147°12'E, 315–2747 m, R/V *Kaiyo-maru*, operation number KOC-17 A, station 28B, KOC net, 0835–1700 hrs, 11 June 1982. • NSMT-P 57245 (formerly TH 831309), 289.7 mm SL, northwestern Pacific, 23°N, 150°E, 2305–2631 m, R/V *Kaiyo-maru*, operation number 83KMT6, station S., KMT net, 1 June 1983, coll. E. Fujii. • OS 14102, 152.7 mm SL, northeastern Pacific, 39°35'24"N, 127°53'36"W, 2212–2350 m, 11 Dec. 1981.

*Gyrinomimus notius* (= *G. grahami*). Holotype: • ZIN 46010, 173.6 mm SL, Ross Sea, Antarctic Ocean, 72°26'S, 175°11'E, depth 445–470 m, R/V *Mis Unoni*, pelagic otter trawl, 8 Mar. 1981.

*Gyrinomimus parri*. Holotype: • USNM 196180, 41.2 mm SL, Gulf of Mexico, 26°52'12.0"N, 89°43'48.0"W, 2469 m, R/V *Oregon*, cruise 60, station 2573, 40-ft Flat Trawl, 28 July 1959. Non-type: • AMS I.20314-010, 147.9 mm SL, 100 km east of Broken Bay, New South Wales, Australia, 33°28'S, 152°34'E–33°36'S, 152°35'E, 5–650 m over bottom depth of 3658–3840 m, FRV *Kapala*, K 77-24-10, Engel midwater trawl, 0530–1045 hrs, 14 Dec. 1977, coll. J. Paxton et al.

*Gyrinomimus simplex* (= *G. myersi*). Holotype: • YPM 1218, 66 mm SL, northwestern Gulf of Mexico, 26°00'N, 93°32'W, ca 1200 m over bottom depth of 2615–2789 m, Atlantis station 2853.

## Supplementary Material

XML Treatment for Gyrinomimus

XML Treatment for Gyrinomimus alepis

XML Treatment for Gyrinomimus amaokai

XML Treatment for Gyrinomimus johnpaxtoni

XML Treatment for Gyrinomimus johnsoni

## Additional information

### Artificial Intelligence (AI) use

The authors accept full responsibility for the content of the manuscript, including the disclosure of any use of AI.

The following AI tools were used in the preparation of this manuscript:

**AI tools**: ChatGPT.

**Used for**: Language, style and writing.

**Description**: AI tools were used to correct grammar errors and wordings. All sentences were originally written by the authors.

### Author ORCIDs

Y. Su https://orcid.org/0000-0002-3576-9229

H.-C. Ho https://orcid.org/0000-0003-1154-601X

### Data availability

All of the data that support the findings of this study are available in the main text.
